# MCHIME-Det: A Lightweight Visual Sensing Framework with Multi-Scale Context and Memory Enhancement for Classroom Behavior Detection

**DOI:** 10.3390/s26216922

**Published:** 2026-10-31

**Authors:** Ting Zhang, Zhaowen Chen, Yingchao Wang, Kun Yang, Yulun Chen, Feng Xue, Tianfu Li, Zhen Sun, Wei Wang, Hong Zhang

**Affiliations:** 1 School of Educational Science, Hunan Normal University, Changsha 410081, China; 2 Key Laboratory of Nondestructive Testing, Fujian Polytechnic Normal University, Fuqing 350300, China; 3 School of Intelligent Manufacturing and Electrical Engineering, Xinjiang College of Science & Technology, Korla 841000, China; 4 Graduate School of Information, Production and Systems, Waseda University, Kitakyushu 808-0135, Japan; 5 Graduate School of Information Science and Technology, The University of Tokyo, Tokyo 113-8654, Japan; 6 School of Civil Engineering, Southeast University, Nanjing 211189, China; 7 Department of Civil and Environmental Engineering, University of Wisconsin–Madison, Madison, WI 53706, USA

**Keywords:** edge AI, visual sensing, classroom behavior detection, lightweight object detection, visual state-space model, hierarchical feature fusion, memory-augmented representation, cross-source evaluation

## Abstract

Camera-based visual sensing for classroom behavior detection remains challenging because fine-grained behaviors often differ only in subtle posture and hand–object cues, while dense seating, occlusion, scale variation, and background interference further weaken discriminative visual evidence. To address these issues, this study proposes the Multi-scale Context, Hierarchical Integration, and Memory Enhancement Detector (MCHIME-Det), a lightweight, multi-scale context and memory-enhanced detector built on YOLO26n. The framework introduces three complementary components: C3k2PMM combines multi-scale local modeling with direction-aware selective state-space scanning; CGHFF performs channel-guided hierarchical feature interaction across adjacent feature levels; and C2MECM uses input-dependent memory experts to compensate for ambiguous high-level representations. For evaluation, two public classroom behavior datasets were combined into a corpus containing 11,879 images and 71,657 annotated instances across three categories: hand-raising, reading, and writing. On the curated image-level split, MCHIME-Det achieves 76.3% precision, 74.9% recall, 82.1% mAP_50_, and 61.4% mAP_50:95_, improving the YOLO26n baseline by 5.6 and 4.4 percentage points in mAP_50_ and mAP_50:95_, respectively. These gains are achieved with only 2.36 M parameters and 5.2 GFLOPs, while maintaining 182 FPS on an RTX 4090 and 17.1 FPS on a Jetson Orin Nano edge device. Ablation, cross-architecture, scale-, density-, and overlap-conditioned analyses, cross-source evaluation, image-corruption robustness tests, and cross-hardware benchmarks characterize model performance on the curated benchmark, three sampled external datasets, specified synthetic corruptions, and tested hardware platforms. These results support hierarchical local–global refinement and memory-assisted compensation as effective strategies for lightweight camera-based visual sensing under the evaluated image datasets and inference settings.

## 1. Introduction

### 1.1. Background and Motivation

Classroom behavior provides observable evidence of how students participate in learning activities and interact with instructional environments. Behaviors such as reading, writing, and hand-raising are therefore frequently used as observable indicators in classroom observation and learning-process analysis rather than direct measurements of cognition or learning outcomes [1,2,3,4]. Conventional classroom observation, however, depends heavily on manual recording and expert interpretation, which limits continuous, large-scale, and fine-grained analysis [1].

Computer vision provides a non-contact approach for automated classroom observation [5]. Object detection is particularly suitable because it can localize multiple students while simultaneously assigning behavior categories. Recent classroom-specific studies have shown that lightweight YOLO-based detectors, multi-level feature enhancement, and attention mechanisms can improve real-time recognition in complex classroom scenes [6,7,8,9]. Nevertheless, classroom behavior detection remains substantially more difficult than conventional object detection because behavior classes are often distinguished by subtle posture, hand motion, and human–object interaction cues rather than by large differences in object appearance [3,10].

### 1.2. Challenges in Classroom Behavior Detection

The difficulty of classroom behavior detection can be summarized from three closely related aspects.

First, different student behaviors may exhibit high visual similarity [11]. Reading and writing, for example, can share similar sitting postures, head orientations, and upper-body configurations, while the decisive information may be concentrated in relatively small regions such as the hands, arms, books, or writing area. These local discriminative cues are easily weakened during repeated feature extraction and downsampling, particularly for students located far from the camera [3,10].

Second, classroom scenes contain substantial semantic interference. Desks, chairs, books, computers, neighboring students, and partially visible body regions are strongly correlated with student activities but are not always useful for distinguishing behavior categories. In dense classrooms, features from adjacent students may further interact spatially, making it difficult to preserve behavior-specific representations. Similar density- and occlusion-related difficulties have been reported in recent classroom detectors [7,12,13].

Third, student instances exhibit considerable variation in scale, visibility, and overlap. Students near the camera occupy large image regions, whereas those in the rear of the classroom may contain only limited spatial detail. Occlusion caused by desks, seats, and neighboring students further reduces the visible evidence available for recognition. Small-target and occlusion-aware classroom studies likewise identify these factors as persistent sources of missed or confused detections [14,15,16]. Related monitoring studies in livestock and infrastructure applications have also examined challenging visual recognition conditions [17,18]. Consequently, a detector must preserve fine-grained behavioral information while remaining robust to scale variation, crowding, and partial visibility.

These difficulties indicate that classroom behavior detection is not simply a localization problem. Effective detection requires the progressive preservation and refinement of task-relevant behavioral representations throughout the feature hierarchy [1].

### 1.3. Existing Methods and Research Gap

Deep-learning-based object detectors have increasingly been applied to classroom behavior analysis because of their ability to jointly learn spatial localization and semantic classification [19]. Similar lightweight detection frameworks in other sensing domains have also demonstrated the importance of efficient feature enhancement and cross-view feature integration for recognizing small or weak targets in complex environments [20]. Existing classroom studies have strengthened multi-scale feature representation, attention-based feature selection, and lightweight feature extraction. Representative examples include multi-scale attention-enhanced YOLOv5 and YOLOv8 variants, cognitive-engagement detectors based on lightweight attention, and multi-level classroom behavior networks such as SBD-Net and YOLO-AMM [2,6,7,8,9,10,21].

Long-range contextual modeling has also received increasing attention. SCB-DETR uses multiscale deformable transformer representations for occlusion-resilient student learning behavior detection, whereas SLBDetection-Net combines behavior-aware attention, Transformer encoding, and small-target processing for closed- and open-set classroom scenarios [12,15]. More recently, SLB-Mamba has introduced visual state-space modeling into student learning behavior detection, while YOLO-APPA and DBRA-SCSA-YOLO further emphasize boundary-aware fusion, multi-scale adaptation, and spatial–channel attention for dense and occluded classroom scenes [13,16,22].

Compared with SLB-Mamba’s detector-level visual state-space modeling, C3k2PMM embeds dilation-partitioned local extraction and mask-guided four-direction SS2D within the lightweight C3k2 hierarchy. Compared with the deformable Transformer representation used by SCB-DETR, C3k2PMM preserves a convolutional local branch and a selective scanning branch at multiple backbone and neck resolutions, directly linking fine-grained hand–object evidence with spatially separated posture context [12,22].

CGHFF differs from boundary-aware fusion and spatial–channel attention designs by applying spatial recalibration, reciprocal adjacent-level modulation, and grouped channel refinement in sequence at the P3/8 and P4/16 fusion sites [13,16]. C2MECM extends learnable-memory retrieval and sparse expert routing through input-dependent selection of memory experts with parallel global-pattern and spatial-context retrieval after SPPF. Together, these stage-specific operations form a continuous refinement path for hand-raising, reading, and writing detection [23,24].

Although these studies provide effective solutions to particular aspects of classroom perception, the principal difficulties often occur simultaneously at different representation levels: weak local action cues, high-level semantic interference, and scale-dependent spatial ambiguity. In addition, many published evaluations remain centered on a principal dataset or a limited acquisition setting, so robustness across independent data sources, image degradations, and heterogeneous deployment hardware is less frequently examined in a unified experimental framework [1]. These observations motivate a lightweight detector that integrates complementary local–global feature modeling, cross-level feature interaction, and memory-assisted semantic compensation, together with evaluation protocols that explicitly probe transferability and difficult-scene robustness.

### 1.4. Proposed Framework and Contributions

To address the above challenges, this study develops a lightweight classroom behavior detection framework with hierarchical feature refinement. The framework progressively strengthens behavior-discriminative information through stage-specific local–global extraction, adjacent-level interaction, and memory-assisted semantic compensation across the feature hierarchy. It is designed to preserve subtle local behavioral cues, suppress task-irrelevant semantic interference, and improve spatial discrimination under scale variation, occlusion, and inter-student interference, while maintaining a lightweight detection architecture suitable for efficient classroom analysis.

For evaluation, two publicly available classroom behavior datasets, SCB-2025-7-public-1 and SCB-Dataset3-S-2, are combined under a common three-class annotation space comprising hand-raising, reading, and writing. The two sources provide varied classroom layouts, student distributions, target scales, occlusion patterns, and visual conditions.

**Figure 1:**
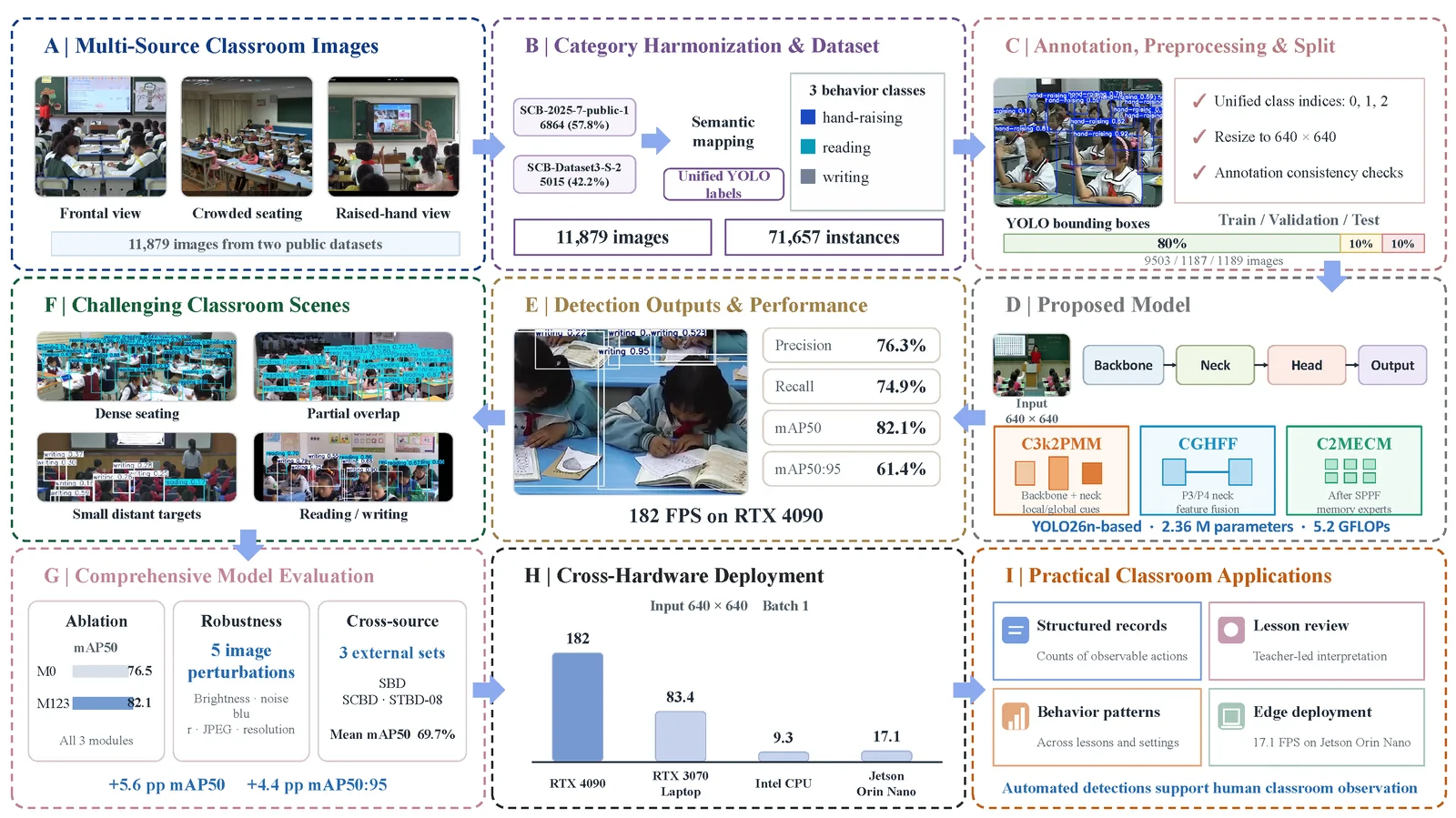
Overall framework of the proposed classroom behavior detection system, including multi-source dataset construction, category harmonization and preprocessing, the proposed lightweight detection model, challenging-scene analysis, comprehensive evaluation, cross-hardware inference benchmarking, and potential classroom applications.

The overall workflow of the proposed framework is illustrated in Figure 1. It integrates multi-source data construction and category harmonization, annotation and preprocessing, hierarchical feature refinement, challenging-scene detection, comprehensive model evaluation, cross-hardware deployment, and practical classroom applications within a unified pipeline.

The main contributions of this study are summarized as follows:

- A lightweight classroom behavior detector with hierarchical feature refinement is developed to address three major task-specific challenges: weak local behavioral cues, semantic interference from classroom context and neighboring students, and scale-dependent spatial ambiguity. The proposed framework progressively enhances behavior-discriminative representations while maintaining low computational complexity. More specifically, C3k2PMM targets fine-scale hand–object evidence and longer-range body context, CGHFF combines spatial detail with intermediate-level semantic cues, and C2MECM provides memory-based compensation when high-level behavior evidence is incomplete.
- Comprehensive experiments are conducted to evaluate the proposed framework from multiple perspectives, including component ablation, comparison with representative general-purpose and classroom-specific detectors, class-wise performance, scale- and overlap-conditioned analysis, robustness evaluation, computational efficiency, and model-level cross-hardware inference benchmarking. The experimental results characterize detection accuracy on the curated image benchmark, performance under the specified synthetic corruptions, and model-level inference throughput on the evaluated hardware.

## 2. Multi-Source Classroom Behavior Dataset

### 2.1. Data Sources

The classroom behavior dataset used in this study was assembled exclusively from two publicly available classroom behavior datasets, SCB-2025-7-public-1 and SCB-Dataset3-S-2. Both are associated with the SCB classroom behavior dataset [25] and provide bounding-box annotations of student behaviors in realistic classroom scenes. Because the two public sources belong to the same broader dataset family, particular attention was paid to annotation consistency, image duplication, and potentially highly similar frames during dataset integration and curation.

The present study focuses on three representative classroom behaviors: hand-raising, reading, and writing. These behaviors are frequently observed during classroom learning activities but exhibit different levels of visual separability. In particular, reading and writing often involve similar seated postures and upper-body configurations, whereas hand-raising generally exhibits more distinctive gesture characteristics.

Following duplicate removal and data curation, the final dataset contained 11,879 images, including 6864 images from SCB-2025-7-public-1 and 5015 images from SCB-Dataset3-S-2. These numbers describe the final retained corpus after source filtering and data curation. No self-collected images were included.

### 2.2. Category Harmonization, Data Curation, and Evaluation Split

The label archives of both public datasets contain only the three retained behavior classes. Their annotations were therefore integrated under a unified three-class detection task consisting of hand-raising, reading, and writing.

For consistency across the two sources, the final class indices were defined as 0 for hand-raising, 1 for reading, and 2 for writing. The annotation files were checked to ensure consistent class indices and bounding-box representations, after which all retained samples were organized in a unified YOLO detection format.

Data curation was performed before establishing the final training, validation, and test partitions. Exact duplicate images identified in the collected source archives were removed, and the retained images formed the final corpus of 11,879 images and 71,657 annotated instances. The curated corpus was then organized into training, validation, and test subsets containing 9503, 1187, and 1189 images, respectively. The training subset was used for model optimization, the validation subset for model selection and parameter adjustment, and the test subset for final quantitative comparison.

To further reduce potential leakage caused by highly similar classroom frames, additional screening was performed for near-duplicate images and visually adjacent frames. Images that could be identified as near-duplicates or as originating from the same source sequence or classroom session were kept within the same subset rather than distributed across the training, validation, and test partitions. Consequently, no identified exact duplicate or identifiable near-duplicate frame group was retained across different subsets.

The released metadata do not provide complete video-, classroom-, camera-, or sequence-level provenance for every image. The resulting training, validation, and test partition is a curated image-level benchmark with exact- and near-duplicate screening; sequence-, classroom-, camera-, and session-level independence remains unverified.

All baseline models, comparison detectors, ablation variants, and the proposed model were trained and evaluated using the same curated partitions and experimental protocol. The main test set supports controlled comparison of detector architectures under this common benchmark protocol. The cross-source experiments in Section 4.7 separately compare model performance on three sampled public source distributions. A sequence-, classroom-, or camera-grouped evaluation requires complete recording-level provenance information for all source images.

### 2.3. Dataset Characteristics

**Figure 2:**
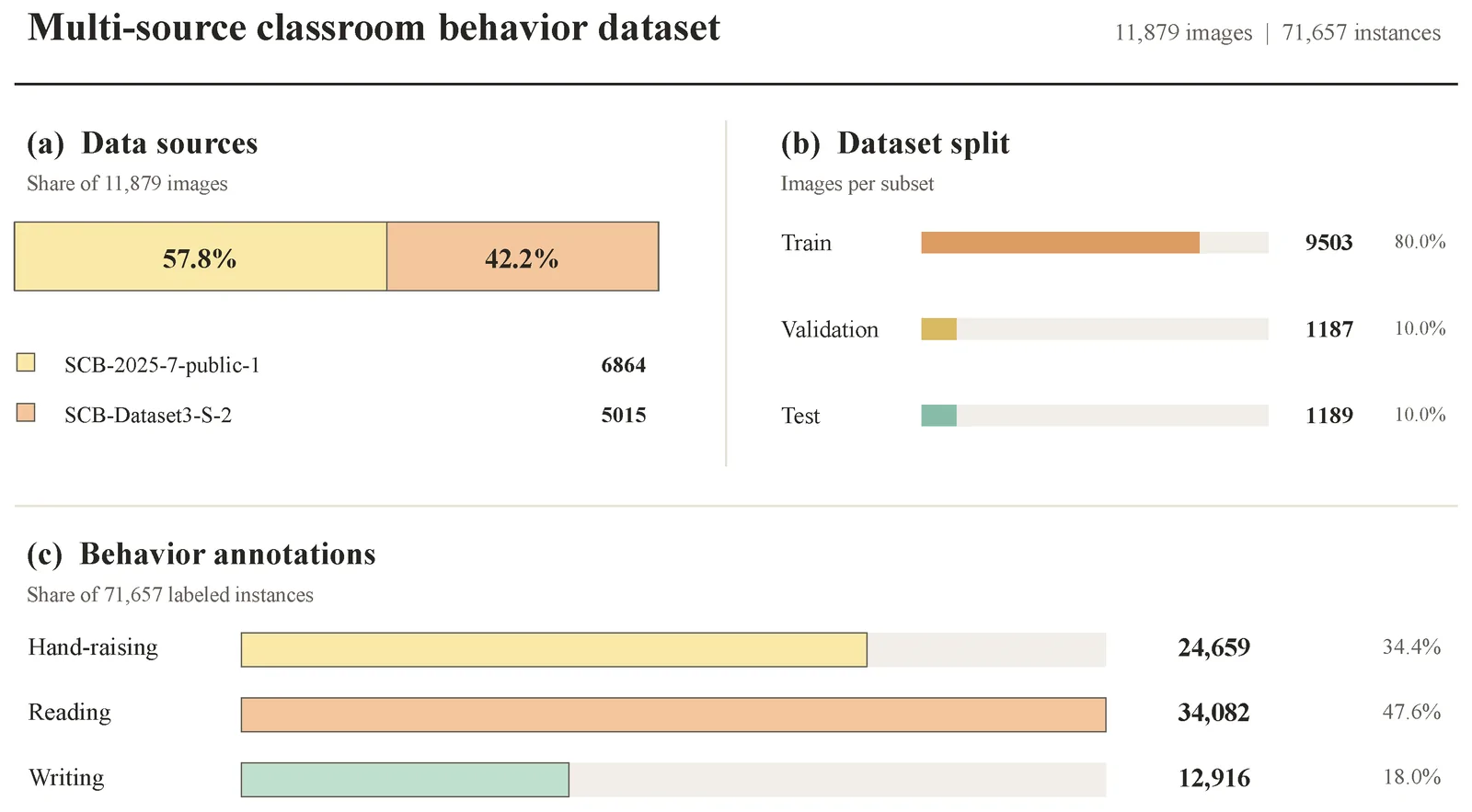
Overview of the combined public classroom behavior dataset, including (**a**) image composition from the two sources; (**b**) training, validation, and test splits; and (**c**) distribution of annotated behavior instances.

The final two-source public dataset contains 11,879 classroom images and 71,657 annotated student behavior instances. Among them, 9503 images were used for training, 1187 for validation, and 1189 for testing, corresponding to approximately 80%, 10%, and 10% of the complete dataset, respectively. Throughout the remainder of this paper, the term “test set” refers specifically to the held-out test subset of this curated benchmark split. Figure 2 provides an overview of the data-source composition, dataset split, and behavior-class distribution.

Table 1 summarizes the image composition of the two public sources after data curation. SCB-2025-7-public-1 contributes 6864 images (57.8%), whereas SCB-Dataset3-S-2 contributes 5015 images (42.2%).

Table 2 presents the image and instance distribution across the training, validation, and test subsets. The complete dataset contains 24,659 hand-raising, 34,082 reading, and 12,916 writing instances, corresponding to 34.4%, 47.6%, and 18.0% of all annotations, respectively. Reading is the most frequent behavior, whereas writing contains fewer samples, resulting in a moderate class imbalance within the dataset.

On average, each image contains approximately 6.0 annotated student behavior instances. The dataset includes both relatively sparse classroom scenes and crowded scenes containing multiple simultaneously visible students. The detection task therefore involves substantial variation in student density, target scale, spatial overlap, visible body regions, and occlusion.

The visual distinction between reading and writing is particularly challenging because both behaviors are typically characterized by seated postures and similar head and upper-body orientations. Their discrimination therefore depends on relatively fine-grained visual cues around the hands, arms, books, and desktop regions. In contrast, hand-raising generally presents a more distinctive pose, although recognition can still become difficult for small targets, partially occluded students, or densely populated classroom scenes.

Overall, the curated dataset provides a unified three-class benchmark for controlled comparison of classroom behavior detectors. The main experiments therefore focus on relative model performance under identical data partitions and training conditions. The complementary cross-source experiments assess transfer across three evaluated public source distributions; conclusions about unseen recording sessions, cameras, or student populations depend on verified source-level independence.

## 3. Proposed Classroom Behavior Detection Framework

### 3.1. Overall Architecture

**Figure 3:**
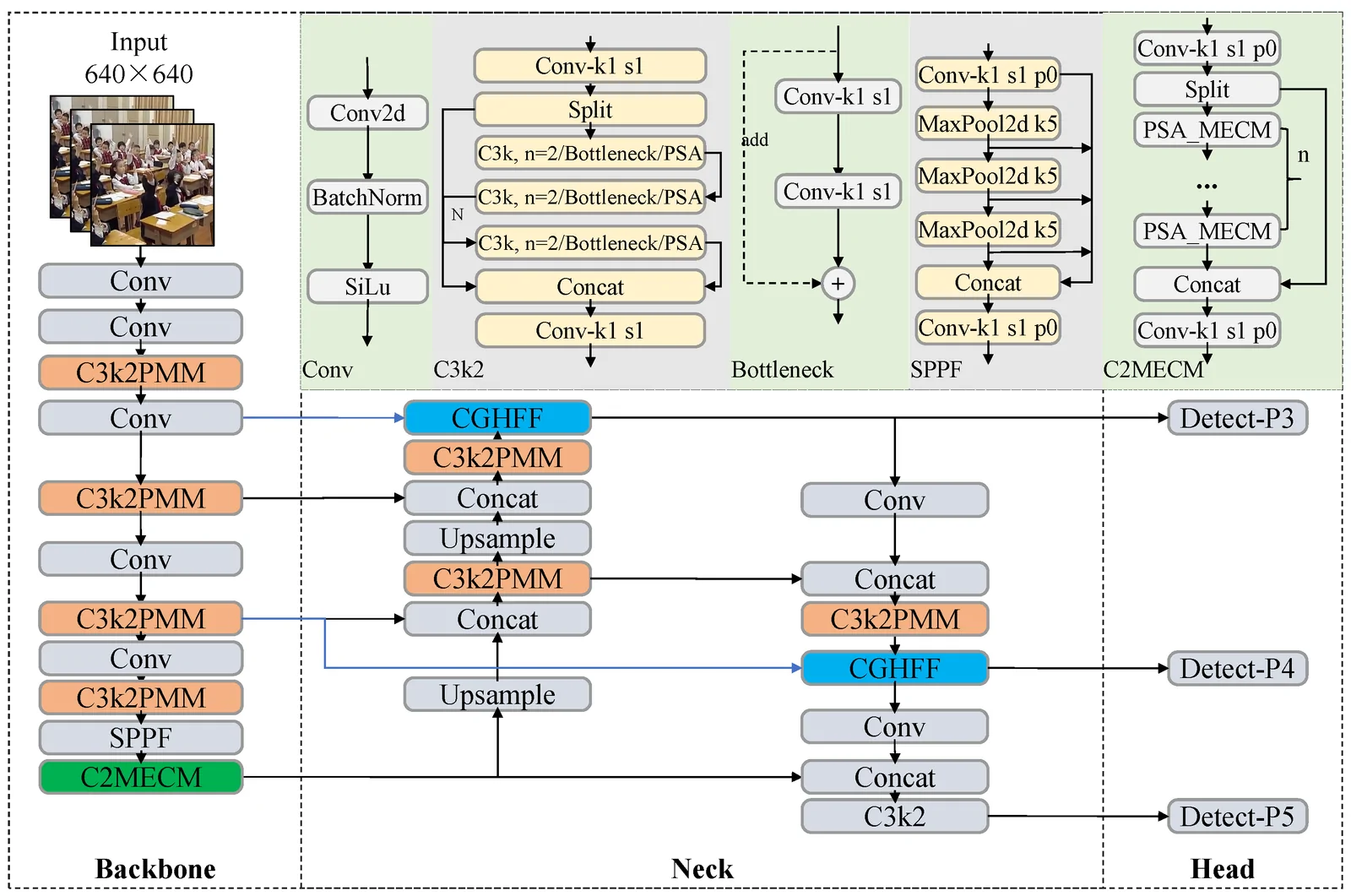
Overall architecture of MCHIME-Det. C3k2PMM enhances multi-scale local–global spatial representation, CGHFF performs channel-guided hierarchical feature fusion, and C2MECM introduces memory-assisted semantic compensation.

The proposed classroom behavior detector is developed from YOLO26n and retains its backbone–neck–head organization and three detection branches at P3, P4, and P5. As shown in Figure 3, three components are introduced to improve the representation of hand-raising, reading, and writing: C3k2PMM, CGHFF, and C2MECM.

C3k2PMM replaces selected C3k2 blocks across the backbone and neck so that multi-scale local extraction and directional long-range modeling are applied at multiple feature resolutions. CGHFF is introduced at the two principal cross-level fusion positions associated with the P3/8 and P4/16 pathways, where higher-resolution spatial detail and stronger intermediate semantics are combined. C2MECM is placed at the deepest semantic stage after SPPF so that memory retrieval and expert routing operate on compact, high-level behavior representations rather than early low-level textures. The resulting P3, P4, and P5 features are finally forwarded to the original detection heads.

### 3.2. Pyramid Mask Mamba Feature Extraction Module

The original C3k2 block mainly relies on convolutional transformations with limited effective receptive fields. Multi-scale representation has long been important for preserving object evidence across spatial resolutions, as illustrated by multi-scale backbone designs such as Res2Net [26]. In parallel, selective state-space models provide a linear-complexity mechanism for long-range dependency modeling [27]; visual state-space approaches extend these ideas to image representation learning [28]. Related lightweight detection studies have also demonstrated the effectiveness of jointly modeling global–local representations and directional information for object detection in complex monitoring environments [29,30,31,32]. Beyond generic visual representation learning, Mamba-based modeling has also been combined with super-resolution reconstruction and YOLO-family detection for weak-target recognition in challenging sensing imagery  [33,34]. For classroom behavior detection, discriminative information may involve spatially separated regions, such as the arm and torso in hand-raising or the hand, book, and desk in reading and writing. Motivated by these complementary local and long-range modeling principles, a PMM-style local–global transformation is integrated into the C3k2 hierarchy by replacing the original C3k transformation, forming C3k2PMM. The resulting C3k2PMM design couples dilated local representations with directionally masked state-space scanning inside lightweight C3k2 blocks.

**Figure 4:**
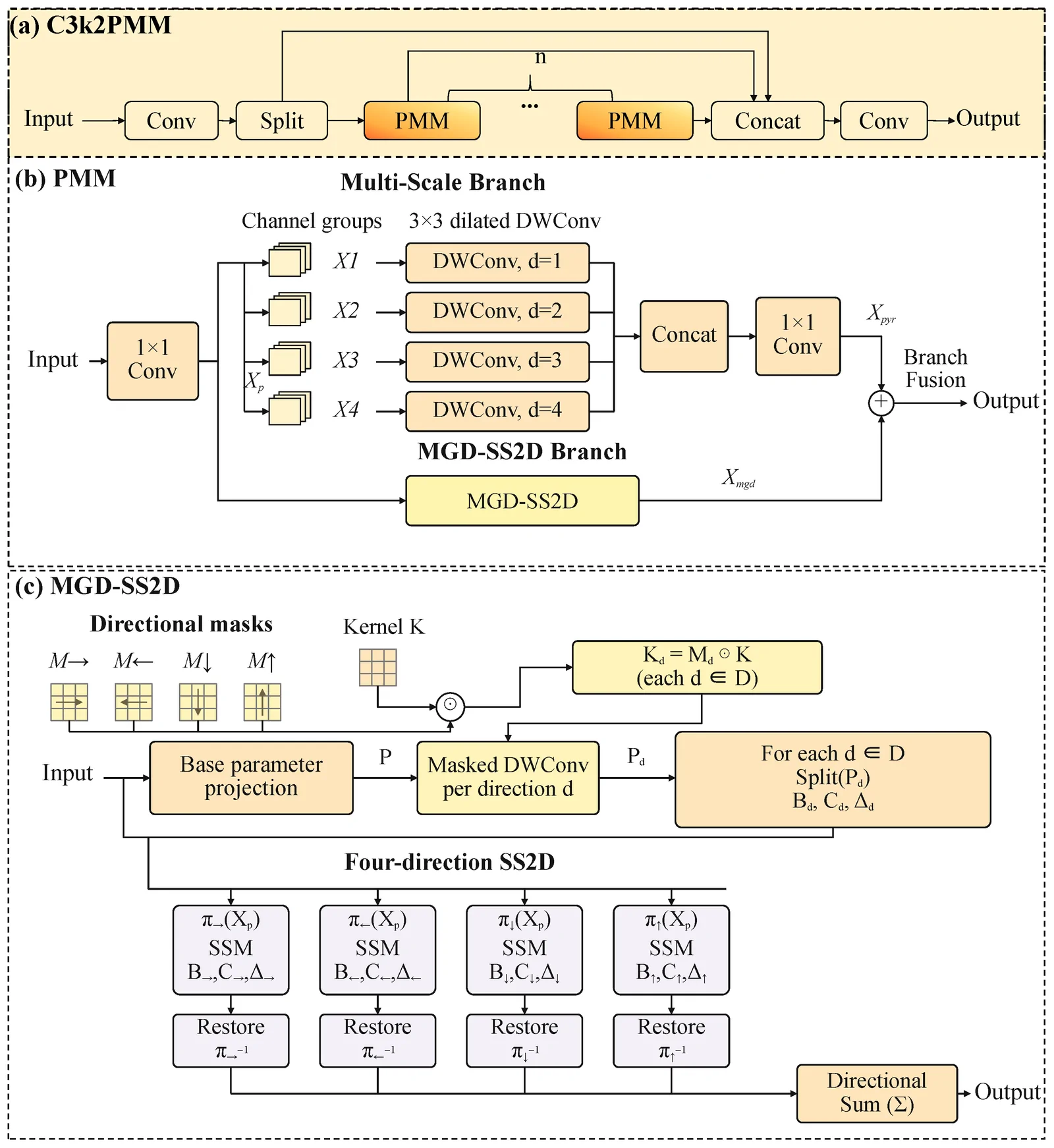
Structure of the proposed C3k2PMM module. The upper panel enlarges the C3k2PMM and PMM data paths, while the lower panel enlarges the MGD-SS2D directional scanning mechanism and operation symbols.

As shown in Figure 4, C3k2PMM retains the original split–transform–concatenate structure. Given an input feature $X\in R^{B\times C\times H\times W}$, a channel projection is first performed:

(1) $$X_{p}={\mathrm{Conv}}_{1\times 1}\left(X\right).$$

PMM contains two parallel branches. In the multi-scale branch, $X_{p}$ is divided into *n* channel groups,

(2) $$X_{p}=\left[X_{1},X_{2},\ldots ,X_{n}\right],$$

and each group is processed using a 3×3 depthwise convolution with a different dilation rate $d_{i}$:

(3) $${\tilde{X}}_{i}={\mathrm{DWConv}}_{3\times 3}^{d_{i}}\left(X_{i}\right),\;i=1,\ldots ,n.$$

The implementation uses four channel groups with dilation rates of 1, 2, 3, and 4, respectively, to capture complementary local receptive fields.

The multi-scale representation is obtained by

(4) $$X_{\mathrm{pyr}}={\mathrm{Conv}}_{1\times 1}\left([{\tilde{X}}_{1},{\tilde{X}}_{2},\ldots ,{\tilde{X}}_{n}]\right).$$

The second branch employs Mask-Guided Directional Selective Scan 2D (MGD-SS2D). This branch applies directional masking across four spatial traversal routes to incorporate classroom-specific spatial structure. The base parameters are generated as

(5) $$P={\mathrm{Conv}}_{1\times 1}\left({\mathrm{DWConv}}_{3\times 3}\left(X_{p}\right)\right),$$

For each scanning direction d∈D={→,←,↓,↑}, the learnable depthwise kernel *K* is modulated by a direction-specific mask $M_{d}$:

(6) $$K_{d}=M_{d}⊙K,$$

where ⊙ denotes element-wise multiplication. The four masks are fixed, non-trainable 3×3 buffers. They are generated from the left-to-right base template by horizontal reversal, transposition, and vertical reversal:

(7) $$\begin{array}{c} M_{\to }=\left[\begin{array}{ccc} 1.5 & 1.3 & 1.0\\ 1.5 & 1.3 & 1.0\\ 1.5 & 1.3 & 1.0 \end{array}\right],\;M_{\leftarrow }={\mathrm{Flip}}_{W}\left(M_{\to }\right),\\ M_{↓}=M_{\to }^{T},\;M_{↑}={\mathrm{Flip}}_{H}\left(M_{↓}\right). \end{array}$$

Each mask is broadcast over the channels of *K*, while *K* itself remains trainable. The masked depthwise convolution is executed independently for each direction before the state-space parameters are generated:

(8) $$\begin{array}{cc} P_{d} & ={\mathrm{DWConv}}_{3\times 3}\left(P;K_{d}\right),\\ \left(B_{d},C_{d},\Delta_{d}\right) & =\mathrm{Split}(P_{d}),\;d\in D. \end{array}$$

Accordingly, the four routes share the projected feature *P* and the underlying trainable kernel *K*, but use distinct masked kernels and distinct parameter tensors $\left(B_{d},C_{d},\Delta_{d}\right)$. The traversal operators use row-major order, its reverse, row-major order after swapping the spatial axes, and the reverse of that transposed order, respectively:

(9) $$\begin{array}{cccc} \pi_{\to }\left(X_{p}\right) & ={\mathrm{Vec}}_{\mathrm{row}}\left(X_{p}\right),\; & \pi_{\leftarrow }\left(X_{p}\right) & =\mathrm{Rev}(\pi_{\to }\left(X_{p}\right)),\\ \pi_{↓}\left(X_{p}\right) & ={\mathrm{Vec}}_{\mathrm{row}}\left(T_{HW}\left(X_{p}\right)\right),\; & \pi_{↑}\left(X_{p}\right) & =\mathrm{Rev}(\pi_{↓}\left(X_{p}\right)). \end{array}$$

After selective scanning, $\pi_{d}^{-1}$ restores each sequence to the original H×W layout, and the four restored outputs are combined by element-wise summation:

(10) $$X_{\mathrm{mgd}}=\sum_{d\in D}\pi_{d}^{-1}\left(\mathrm{SSM}\left(\pi_{d}\left(X_{p}\right);B_{d},C_{d},\Delta_{d}\right)\right).$$

Directional masking and selective scanning enable information propagation over a wider spatial range, which is useful for elongated arms, body structures, and book boundaries.

The final PMM feature is obtained by

(11) $$X_{\mathrm{PMM}}=X_{\mathrm{pyr}}+X_{\mathrm{mgd}}.$$

Thus, C3k2PMM combines multi-scale local structures with direction-aware long-range dependencies. In the proposed network, it replaces seven original C3k2 blocks across the backbone and neck, allowing the same local–global refinement principle to be applied before and during multi-scale feature aggregation rather than only at a single terminal stage.

### 3.3. Channel-Guided Hierarchical Feature Fusion Module

Hierarchical feature fusion is a central strategy for multi-scale detection. FPN established top-down feature pyramids with lateral connections, PANet strengthened bottom-up information propagation, and EfficientDet introduced weighted bidirectional feature fusion for efficient multi-scale aggregation [35,36,37]. Attention mechanisms such as squeeze-and-excitation, CBAM, and coordinate attention have further shown that channel and spatial responses can be selectively recalibrated with modest computational overhead [38,39,40,41]. Related camera–radar detection work has also studied interaction between complementary feature representations [42].

Direct concatenation of low- and high-level features, however, may propagate background responses together with useful behavior information. CGHFF therefore combines spatial recalibration and reciprocal cross-level modulation with grouped channel-wise self-attention, adapting these mechanisms to the hierarchical fusion of classroom behavior features. The resulting CGHFF design applies spatial recalibration before reciprocal cross-level feature interaction and grouped channel refinement.

**Figure 5:**
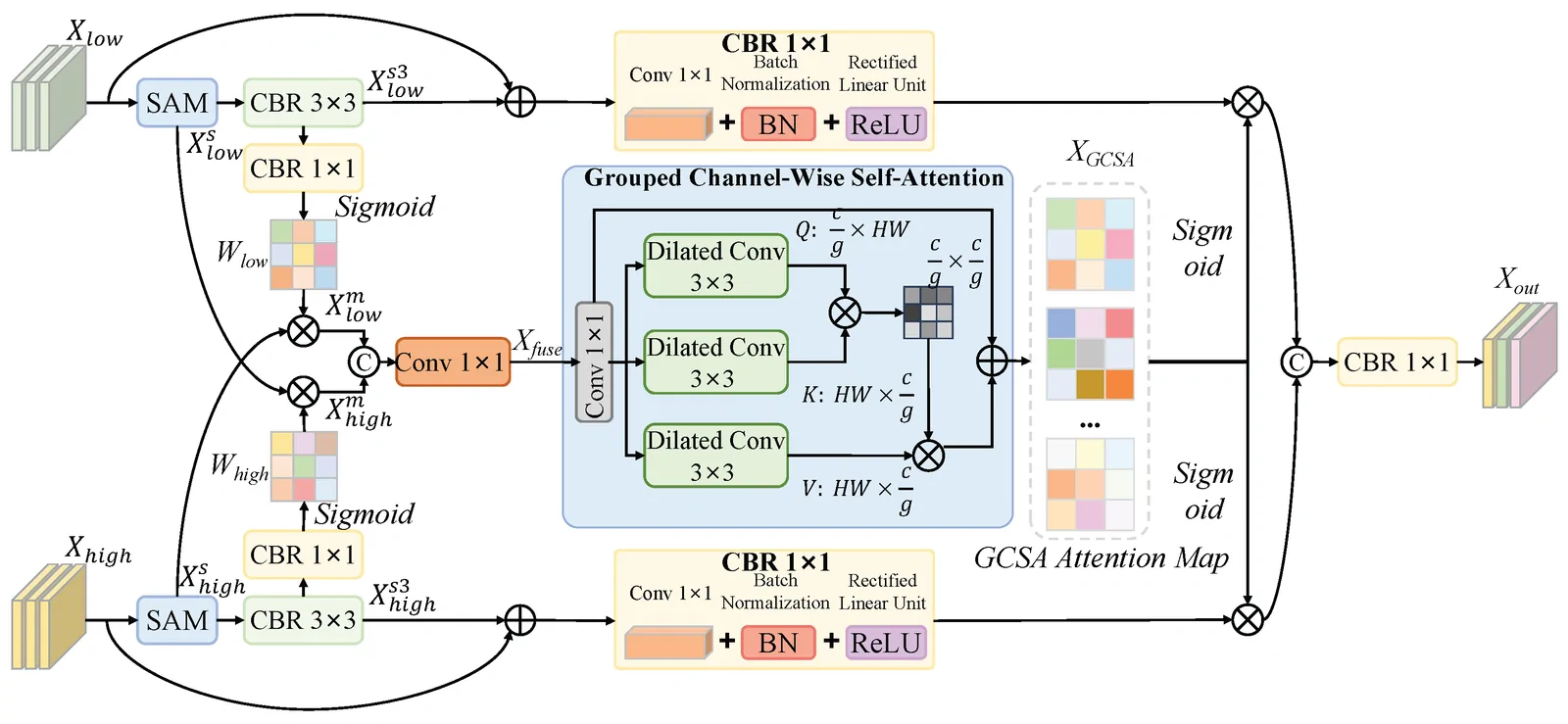
Structure of the proposed CGHFF module. Spatial recalibration and reciprocal cross-level modulation are followed by grouped channel-wise self-attention for adaptive hierarchical feature fusion.

As shown in Figure 5, given a low-level feature $X_{\mathrm{low}}$ and a high-level feature $X_{\mathrm{high}}$, the latter is first upsampled:

(12) $${\bar{X}}_{\mathrm{high}}=\mathrm{Up}\left(X_{\mathrm{high}}\right).$$

Spatial attention is independently applied to both feature levels:

(13) $$X_{\mathrm{low}}^{s}=\mathrm{SAM}\left(X_{\mathrm{low}}\right),\;X_{\mathrm{high}}^{s}=\mathrm{SAM}\left({\bar{X}}_{\mathrm{high}}\right).$$

The spatial attention modules use a 7-by-7 convolutional kernel.

Cross-level weights are then generated through lightweight convolutional transformations:

(14) $$W_{\mathrm{low}}=\sigma \left({\mathrm{CBR}}_{1\times 1}\left(X_{\mathrm{low}}^{s}\right)\right),\;W_{\mathrm{high}}=\sigma \left({\mathrm{CBR}}_{1\times 1}\left(X_{\mathrm{high}}^{s}\right)\right),$$

where CBR denotes convolution, batch normalization, and ReLU activation.

The two feature levels are reciprocally modulated:

(15) $${\tilde{X}}_{\mathrm{low}}=X_{\mathrm{low}}^{s}⊙W_{\mathrm{high}},\;{\tilde{X}}_{\mathrm{high}}=X_{\mathrm{high}}^{s}⊙W_{\mathrm{low}}.$$

The modulated features are concatenated to obtain

(16) $$X_{\mathrm{fuse}}={\mathrm{Conv}}_{1\times 1}\left([{\tilde{X}}_{\mathrm{low}},{\tilde{X}}_{\mathrm{high}}]\right).$$

CGHFF further employs Grouped Channel-Wise Self-Attention (GCSA). Query, key, and value features are generated from $X_{\mathrm{fuse}}$ and divided into *h* channel groups. For the *i*-th group,

(17) $$A^{\left(i\right)}=\mathrm{Softmax}\left(\frac{Q^{\left(i\right)}{\left(K^{\left(i\right)}\right)}^{T}}{\sqrt{d}}\right),\;Y^{\left(i\right)}=A^{\left(i\right)}V^{\left(i\right)},$$

where *d* denotes the channel dimension of each group. GCSA uses four channel groups.

The group responses are concatenated and projected to obtain the channel attention representation:

(18) $$X_{\mathrm{GCSA}}={\mathrm{Conv}}_{1\times 1}\left([Y^{\left(1\right)},Y^{\left(2\right)},\ldots ,Y^{\left(h\right)}]\right).$$

The final channel guidance is

(19) $$W_{c}=\sigma \left(X_{\mathrm{GCSA}}\right),$$

and the output feature is obtained by

(20) $$X_{\mathrm{out}}={\mathrm{CBR}}_{1\times 1}\left([W_{c}⊙{\tilde{X}}_{\mathrm{low}},W_{c}⊙{\tilde{X}}_{\mathrm{high}}]\right).$$

CGHFF is deployed at the P3/8 and P4/16 pathways because these are the two principal neck locations where adjacent-resolution features are fused. The P3/8 pathway retains finer spatial evidence for small or distant students, whereas the P4/16 pathway provides stronger intermediate-level semantics. Restricting CGHFF to these fusion sites avoids repeatedly applying the same attention operation throughout the neck.

### 3.4. Memory Expert Compensation Module

The original high-level attention mechanism mainly models information within the current image. Learnable memory modules provide a related mechanism for retrieving representative feature prototypes, while sparse mixture-of-experts models demonstrate the utility of input-dependent expert routing [23,24]. Building on these memory-retrieval and expert-routing principles, C2MECM adapts memory-expert compensation to the high-level C2PSA pathway for classroom behavior detection. Classroom behaviors may provide incomplete or ambiguous evidence because of occlusion, limited resolution, and high visual similarity; the memory experts are therefore used to retrieve representative cross-image patterns for the current high-level feature.

**Figure 6:**
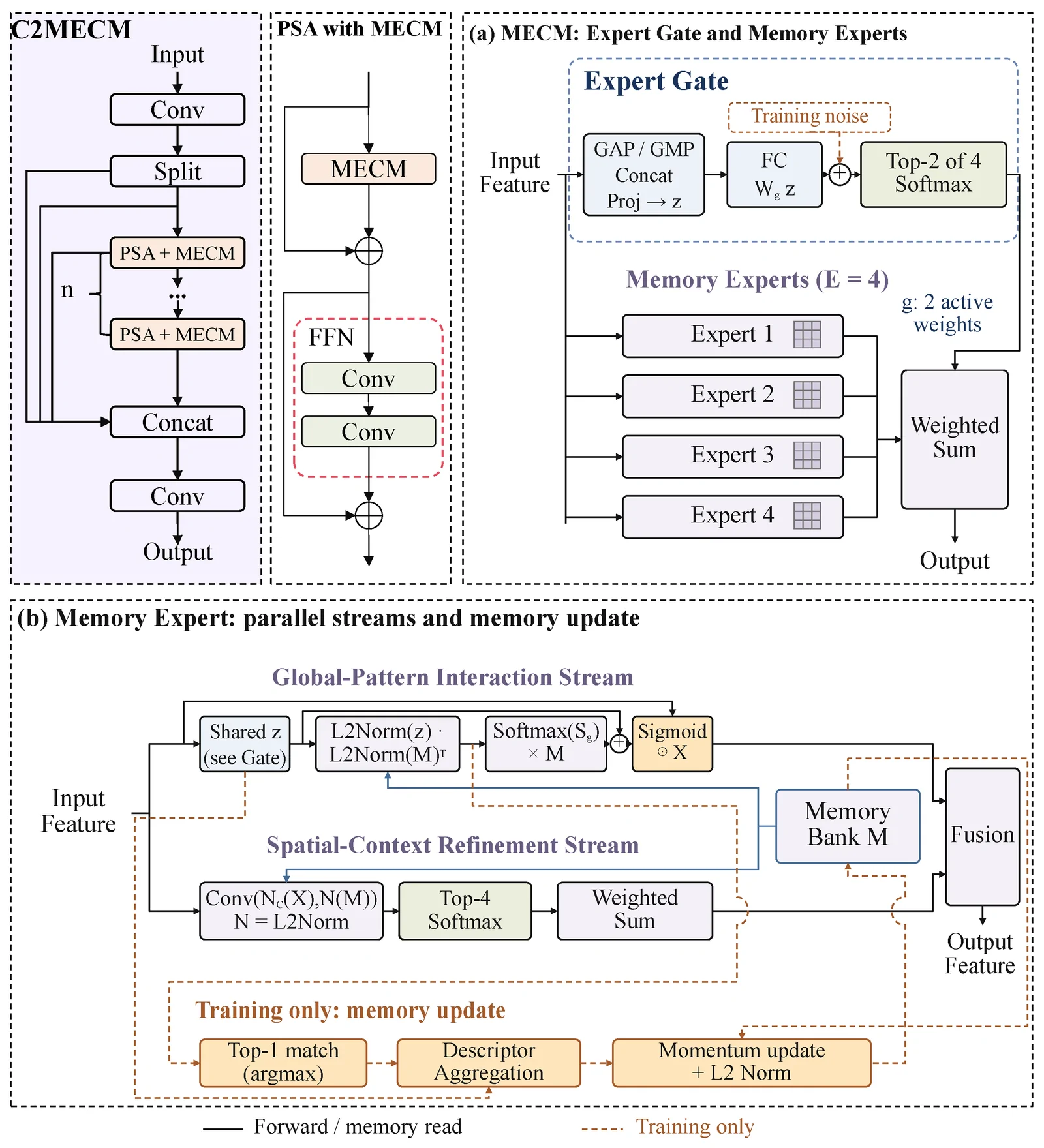
Structure of the proposed C2MECM module. The upper panel enlarges the C2MECM and PSA_MECM overview, while the lower panel enlarges the Expert Gate, Memory Expert, GPStream, and SCStream paths.

As shown in Figure 6, C2MECM retains the overall split and cross-stage structure while incorporating MECM into the high-level feature processing pathway. MECM consists of an Expert Gate, multiple Memory Experts, and memory banks maintained by the training-time update described below.

Given an input feature $X\in R^{B\times C\times H\times W}$, global average and maximum pooling are first used to obtain a compact descriptor *z*. The Expert Gate predicts the expert weights as

(21) $$g=\mathrm{Softmax}\left[\mathrm{TopK}\left(s\right)\right],\;s=W_{g}z+\epsilon$$

The gate contains four memory experts and activates the two highest-scoring experts for each input. During training, the noisy-routing term is generated as

(22) $$\epsilon =1_{\mathrm{train}}\;\mathrm{Softplus}\left(W_{\epsilon }z\right)⊙\xi ,\;\xi ∼N\left(0,I_{E}\right),$$

where $W_{\epsilon }$ is learned jointly with the gate and E=4. A fresh Gaussian sample is drawn for each training input; at inference, $1_{\mathrm{train}}=0$, so ϵ=0 and routing is deterministic. The Top-*K* operator retains the two selected logits, assigns −∞ to the remaining logits, and the subsequent softmax normalizes the two active expert weights to sum to one.

Let $E_{j}\left(X\right)$ denote the output of the *j*-th memory expert. The expert responses are aggregated as

(23) $$X_{\mathrm{MECM}}=\sum_{j=1}^{E}g_{j}E_{j}\left(X\right),$$

where *E* denotes the total number of experts.

Each expert contains a Global-Pattern Interaction Stream (GPStream) and a Spatial-Context Refinement Stream (SCStream). Let

(24) $$M\in R^{N_{m}\times C}$$

denote the corresponding memory bank containing $N_{m}$ memory entries. Each expert has 32 memory entries. Before training, every entry is sampled independently from $N(0,C^{-1}I_{C})$ and normalized to unit $\ell_{2}$ norm. The four expert banks are initialized independently. Equations below omit the expert index *j* for readability.

For global pattern retrieval, the pooled feature *z* is matched with the memory bank:

(25) $$S_{g}=L2\mathrm{Norm}\left(z\right)\;L2\mathrm{Norm}{\left(M\right)}^{T}$$

and the memory response is

(26) $$R_{g}=\mathrm{Softmax}\left(S_{g}\right)M.$$

The resulting global compensation is applied as

(27) $$X_{g}=X⊙\sigma \left(z+R_{g}\right).$$

For spatial-context refinement, the memory entries are reshaped as convolutional kernels and matched with the spatial feature:

(28) $$S_{s}=\mathrm{Conv}\left(L2{\mathrm{Norm}}_{C}\left(X\right),L2\mathrm{Norm}\left(M\right)\right)$$

where $S_{s}$ describes the similarity between each spatial location and the memory entries.

For each spatial position *p*, the Top-*K* memory responses are selected and normalized:

(29) $$\alpha_{p,k}=\mathrm{Softmax}\left(\mathrm{TopK}(S_{s}\left(p,:\right))\right),$$

and the spatial compensation feature is obtained as

(30) $$R_{s}\left(p\right)=\sum_{k=1}^{K}\alpha_{p,k}M_{k}$$

Within SCStream, spatial retrieval selects the four highest-scoring entries per location, separately from the two-expert routing decision.

The two streams provide complementary global and spatial memory information within each expert. The memory bank is updated once per mini-batch from the pooled descriptors of samples routed to each active expert. For sample *b* and active expert *j*, let $q_{b}=L2\mathrm{Norm}\left(z_{b}\right)$ and let $m_{j,n}=L2\mathrm{Norm}\left(M_{j,n}\right)$. The top-1 matched memory entry and its gate-weighted matching score are

(31) $$\begin{array}{cc} s_{b,j,n}=q_{b}^{T}m_{j,n}, & n_{b,j}^{★}=\arg\max_{n}s_{b,j,n},\\ \;\;a_{b,j}=g_{b,j}\mathrm{Softmax}{\left(s_{b,j,:}\right)}_{n_{b,j}^{★}}. & \end{array}$$

For every expert *j* and memory slot *n*, scores from all mini-batch samples assigned to that slot are normalized as

(32) $${\bar{a}}_{b,j,n}=\frac{a_{b,j}\;1\left[n_{b,j}^{★}=n\right]}{\sum_{b^{\prime }}a_{b^{\prime },j}\;1\left[n_{b^{\prime },j}^{★}=n\right]+\varepsilon_{0}},\;\varepsilon_{0}=10^{-6},$$

and the batch-aggregated descriptor and residual are

(33) $${\bar{q}}_{j,n}=\sum_{b}{\bar{a}}_{b,j,n}q_{b},\;r_{j,n}={\bar{q}}_{j,n}-m_{j,n}.$$

A slot receiving at least one assignment is updated independently within its expert by

(34) $$\begin{array}{cc} m_{j,n} & \leftarrow L2\mathrm{Norm}\;\left(\mu m_{j,n}+\left(1-\mu \right){\bar{q}}_{j,n}\right),\;\mu =0.9,\\ \; & =L2\mathrm{Norm}\;\left(m_{j,n}+\left(1-\mu \right)r_{j,n}\right). \end{array}$$

Thus, 0.9 weights the previous memory state and the residual coefficient is 1−μ=0.1. Experts that receive no routed samples and slots that receive no assignment remain unchanged. The noisy gate and memory update are active only during training; inference uses deterministic routing and fixed memory banks.

C2MECM is placed at the deepest semantic stage after SPPF, where the feature map is spatially compact and semantically enriched. This limits memory retrieval and sparse expert routing to the stage where high-level ambiguity is most directly represented, while avoiding repeated memory operations on high-resolution low-level features.

The core module-level operations are summarized in Algorithm 1.
**Algorithm 1** Core implementation of the C3k2PMM, CGHFF, and C2MECM modules**Input:** Stage-specific features $X_{p}$, $\left(X_{l},X_{h}\right)$, and *X*; four expert memory banks ${\left\{M_{j}\right\}}_{j=1}^{4}$ (32 entries each)**Output:** Refined features $X_{\mathrm{PMM}}$, $X_{\mathrm{CGHFF}}$, and $X_{\mathrm{MECM}}$ 1: **C3k2PMM: Multi-scale directional refinement** 2: Split $X_{p}$ into four groups ${\left\{X_{i}\right\}}_{i=1}^{4}$ 3: $X_{\mathrm{pyr}}\leftarrow {\mathrm{Conv}}_{1\times 1}\left({\left[{\mathrm{DWConv}}_{3\times 3}^{d_{i}}\left(X_{i}\right)\right]}_{i=1}^{4}\right)$, $d_{i}\in \left\{1,2,3,4\right\}$ 4: $P\leftarrow {\mathrm{Conv}}_{1\times 1}\left({\mathrm{DWConv}}_{3\times 3}\left(X_{p}\right)\right)$ 5: Define fixed masks ${\left\{M_{d}\right\}}_{d\in D}$ by Equation (7) 6: Construct the four routes ${\left\{\pi_{d}\left(X_{p}\right)\right\}}_{d\in D}$ by Equation (9) 7: **for** 
d∈D 
**do** 8:   $K_{d}\leftarrow M_{d}⊙K$ 9:   $P_{d}\leftarrow {\mathrm{DWConv}}_{3\times 3}\left(P;K_{d}\right)$; $\left(B_{d},C_{d},\Delta_{d}\right)\leftarrow \mathrm{Split}\left(P_{d}\right)$10:   $Y_{d}\leftarrow \pi_{d}^{-1}\left(\mathrm{SSM}\left(\pi_{d}\left(X_{p}\right);B_{d},C_{d},\Delta_{d}\right)\right)$11: **end for**12: $X_{\mathrm{PMM}}\leftarrow X_{\mathrm{pyr}}+\sum_{d\in D}Y_{d}$13: **CGHFF: Hierarchical feature fusion**14: $\left(X_{l}^{s},X_{h}^{s}\right)\leftarrow \left({\mathrm{SAM}}_{7\times 7}\left(X_{l}\right),{\mathrm{SAM}}_{7\times 7}\left(\mathrm{Up}\left(X_{h}\right)\right)\right)$15: $\left(W_{l},W_{h}\right)\leftarrow \left(\sigma \left({\mathrm{CBR}}_{1\times 1}\left(X_{l}^{s}\right)\right),\sigma \left({\mathrm{CBR}}_{1\times 1}\left(X_{h}^{s}\right)\right)\right)$16: $\left({\tilde{X}}_{l},{\tilde{X}}_{h}\right)\leftarrow \left(X_{l}^{s}⊙W_{h},X_{h}^{s}⊙W_{l}\right)$17: $X_{f}\leftarrow {\mathrm{Conv}}_{1\times 1}\left(\left[{\tilde{X}}_{l},{\tilde{X}}_{h}\right]\right)$18: $W_{c}\leftarrow \sigma \left({\mathrm{GCSA}}_{h=4}\left(X_{f}\right)\right)$19: $X_{\mathrm{CGHFF}}\leftarrow {\mathrm{CBR}}_{1\times 1}\left(\left[W_{c}⊙{\tilde{X}}_{l},W_{c}⊙{\tilde{X}}_{h}\right]\right)$20: **C2MECM: Memory-expert compensation**21: z←Proj([GAP(X),GMP(X)])22: **if** training **then**23:   Draw $\xi ∼N(0,I_{4})$; $\epsilon \leftarrow \mathrm{Softplus}(W_{\epsilon }z)⊙\xi$24: **else**25:   ϵ←026: **end if**27: $s\leftarrow W_{g}z+\epsilon$; $I_{e}\leftarrow \mathrm{TopKIdx}\left(s,2\right)$; $g_{I_{e}}\leftarrow \mathrm{Softmax}\left(s_{I_{e}}\right)$28: **for** 
$j\in I_{e}$ 
**do**29:   $R_{g}\leftarrow \mathrm{Softmax}\left(L2\mathrm{Norm}\left(z\right)\;L2\mathrm{Norm}{\left(M_{j}\right)}^{T}\right)M_{j}$30:   $X_{g}\leftarrow X⊙\sigma \left(z+R_{g}\right)$31:   $S_{s}\leftarrow \mathrm{Conv}\left(L2{\mathrm{Norm}}_{C}\left(X\right),L2\mathrm{Norm}\left(M_{j}\right)\right)$32:   **for** each spatial location *p* **do**33:     $I_{p}\leftarrow \mathrm{TopKIdx}\left(S_{s}\left(p,:\right),4\right)$34:     $R_{s}\left(p\right)\leftarrow \sum_{k\in I_{p}}\mathrm{Softmax}{\left(S_{s}\left(p,I_{p}\right)\right)}_{k}M_{j,k}$35:   **end for**36:   $Y_{j}\leftarrow \mathrm{Conv}\left(\left[X_{g},R_{s}\right]\right)$37: **end for**38: $X_{\mathrm{MECM}}\leftarrow \sum_{j\in I_{e}}g_{j}Y_{j}$39: **if** training **then**40:   **for** each active expert *j* and sample *b* routed to *j* **do**41:     $q_{b}\leftarrow L2\mathrm{Norm}\left(z_{b}\right)$; $n_{b,j}^{★}\leftarrow \arg\max_{n}q_{b}^{T}L2\mathrm{Norm}\left(M_{j,n}\right)$42:     $a_{b,j}\leftarrow g_{b,j}\;\mathrm{Softmax}{\left(q_{b}L2\mathrm{Norm}{\left(M_{j}\right)}^{T}\right)}_{n_{b,j}^{★}}$43:   **end for**44:   **for** each expert-slot pair (j,n) receiving at least one assignment **do**45:     Normalize $\left\{a_{b,j}:n_{b,j}^{★}=n\right\}$ across the mini-batch and compute ${\bar{q}}_{j,n}$46:     $m_{j,n}\leftarrow L2\mathrm{Norm}\left(M_{j,n}\right)$; $r_{j,n}\leftarrow {\bar{q}}_{j,n}-m_{j,n}$47:     $M_{j,n}\leftarrow L2\mathrm{Norm}\left(m_{j,n}+0.1r_{j,n}\right)$48:   **end for**49: **end if**50: **return** 
$X_{\mathrm{PMM}},\;X_{\mathrm{CGHFF}},\;X_{\mathrm{MECM}}$

## 4. Experiments

### 4.1. Experimental Setup

Training, validation, ablation, and primary accuracy experiments were conducted on the workstation described below. Cross-hardware inference benchmarks were conducted separately on the four platforms specified in Section 4.9. The workstation was equipped with an NVIDIA GeForce RTX 4090 GPU with 24 GB memory, an Intel Core Ultra 9 285K CPU, and 64 GB RAM. The operating system was Windows 11, and all models were implemented using PyTorch 2.1.0 with CUDA 11.8 and Python 3.10.20. The detailed experimental environment is summarized in Table 3.

For the controlled experiments involving YOLO26n and the proposed variants, the same training configuration was adopted to ensure a fair comparison. Accordingly, the incremental effect of the proposed modules is assessed primarily against the matched YOLO26n baseline, while comparisons across other detector families provide broader performance context. For these controlled experiments, input images were resized to 640×640 pixels, and the models were trained for 200 epochs with a batch size of 16. Stochastic Gradient Descent (SGD) was used as the optimizer with an initial learning rate of 0.001, a final learning-rate factor of 0.01, momentum of 0.937, and weight decay of 0.0005. Eight data-loading workers were used. The complete training configuration is listed in Table 4.

For the representative-detector comparison, every model was retrained on the same curated training partition and evaluated on the same validation and test subsets.RT-DETR-n used COCO-pretrained RT-DETR weights, AdamW with an initial learning rate of $1\times 10^{-4}$ and weight decay of $1\times 10^{-4}$, cosine decay to $1\times 10^{-6}$, 200 epochs, and a batch size of 16. Its augmentation consisted of horizontal flipping, color jitter, and scale jitter between 0.8 and 1.2 at a 640×640 input.

GhostNet-n used an ImageNet-pretrained GhostNet backbone and a randomly initialized detection head. It was trained for 200 epochs with a batch size of 16 using SGD, an initial learning rate of 0.001, a final learning-rate factor of 0.01, momentum of 0.937, and weight decay of 0.0005. Mamba-YOLO-tiny used the COCO-pretrained Mamba-YOLO-T weights and SGD for 300 epochs with a batch size of 16, an initial learning rate of 0.01, cosine decay to 0.0001, momentum of 0.937, and weight decay of 0.0005.

For GhostNet-n and Mamba-YOLO-tiny, the augmentation pipeline included HSV perturbation, random affine transformation, horizontal flipping with probability 0.5, and mosaic augmentation; Mamba-YOLO-tiny additionally used mixup with probability 0.1. Mosaic augmentation was disabled during the final 10 epochs. Both models used 640×640 inputs.

SSD300-MobileNetV2 used an ImageNet-pretrained MobileNetV2 backbone, SGD with an initial learning rate of 0.001, momentum of 0.9, and weight decay of 0.0005, followed by a step schedule with a factor of 0.1 at epochs 80 and 100. It was trained for 120 epochs with a batch size of 32 at 300×300. Its augmentation used photometric distortion, IoU-constrained random cropping, and horizontal flipping with probability 0.5.

Faster R-CNN used the COCO-pretrained ResNet-50-FPN detector, SGD with an initial learning rate of 0.005, momentum of 0.9, and weight decay of 0.0005, with a factor-of-0.1 step decay at epochs 16 and 22. It was trained for 24 epochs with a batch size of 4. Images were resized with the shorter side sampled from 576, 640, and 704 pixels, followed by horizontal flipping with probability 0.5; evaluation used a 640×640 input.

YOLO-Worldv2-n used the released COCO-pretrained YOLO-World-v2 weights and AdamW with an initial learning rate of $2\times 10^{-4}$, weight decay of 0.05, and cosine decay to $2\times 10^{-6}$. It was trained for 100 epochs with a batch size of 16. The augmentation pipeline used mosaic, mixup with probability 0.1, random affine transformation, HSV perturbation, and horizontal flipping with probability 0.5.

YOLOv9t, YOLOv10n, YOLOv11n, YOLOv12n, and YOLOv13n were initialized from their released COCO-pretrained weights and trained for 200 epochs with a batch size of 16. Each used SGD with an initial learning rate of 0.001, a final learning-rate factor of 0.01, momentum of 0.937, weight decay of 0.0005, and a three-epoch warm-up. Their augmentation used mosaic, HSV perturbation, random affine transformation, and horizontal flipping with probability 0.5; mosaic was disabled during the final 10 epochs.

CBPH-Net used its ImageNet-pretrained backbone, AdamW with an initial learning rate of $1\times 10^{-4}$ and weight decay of 0.05, cosine decay to $1\times 10^{-6}$, 200 epochs, and a batch size of 16. SLBDetection-Net used its ImageNet-22K-pretrained Swin backbone with the same AdamW schedule, a five-epoch warm-up, 200 epochs, and a batch size of 8. Both used random resize and crop, horizontal flipping with probability 0.5, and color jitter at 640×640.

All detector families shared the same images, three-class label space, validation-based checkpoint selection, test metrics, and computing platform. No model was tuned on the test subset. The YOLO26n SGD settings in Table 4 were applied only to the matched YOLO26n baseline and MCHIME-Det variants. The model-specific optimizer, learning-rate schedule, training duration, batch size, input resolution, augmentation, and pretraining source used for each comparison detector are reported above.

Model evaluation was performed using the best checkpoint obtained during training. The concrete module settings reported in Section 3—the C3k2PMM dilation rates and scan directions, the CGHFF channel grouping, and the C2MECM expert count, active-expert Top-*K*, memory size, spatial Top-*K*, and memory-update momentum—were selected using the validation subset and fixed before final testing. The validation subset was also used for checkpoint selection, whereas the test subset was reserved for final evaluation and was not used for model selection or hyperparameter tuning. Unless otherwise specified, validation was conducted using the standard Ultralytics evaluation procedure without manually redefining the confidence or IoU thresholds. For the repeatability assessment, all mAP results in the internal-component ablation are reported as mean and standard deviation (SD) over five independent runs under the fixed data partition. Selected mAP results in the principal model comparison and the cross-source evaluations, together with the Baseline and “+All” configurations in the cross-architecture study, are likewise reported as mean and SD over five independent runs under the respective fixed evaluation partitions.

Inference speed was measured separately using a batch size of 1 and an input resolution of 640×640 except for SSD300-MobileNetV2, which used 300-by-300 inputs. Each model was first warmed up for 200 iterations, followed by 1000 timed inference runs. GPU synchronization was performed immediately before and after each forward pass, and gradients were disabled during benchmarking. FPS was calculated from the mean inference latency. Only the network forward pass was timed; image decoding, preprocessing, host-to-device transfers, and post-processing (including NMS) were excluded. The speed-test configuration is summarized in Table 5.

### 4.2. Comparison with Representative Detectors

To evaluate the overall competitiveness of MCHIME-Det, we compared it with representative detectors from different architectural paradigms. The selection covers transformer-based detection, lightweight convolutional backbones, state-space modeling, open-vocabulary detection, two-stage detection, and recent lightweight YOLO generations. The general-purpose methods include RT-DETR-n, GhostNet-n, Mamba-YOLO-tiny, SSD300-MobileNetV2, Faster R-CNN with a ResNet-50 backbone, YOLO-Worldv2-n, and YOLOv9t, YOLOv10n, YOLOv11n, YOLOv12n, YOLOv13n, and YOLO26n. In addition, two classroom-specific detectors with formally published sources were included: CBPH-Net [14] and SLBDetection-Net [15]. YOLO26n serves as the direct architectural baseline, whereas the remaining models provide broader context across detector families. The comparison results are summarized in Table 6.

As shown in Table 6, MCHIME-Det achieves the highest Precision, Recall, mAP_50_, and mAP_50:95_ among the reported comparisons, reaching 76.3%, 74.9%, 82.1%, and 61.4%, respectively. Compared with the YOLO26n baseline, MCHIME-Det improves these four metrics by 7.6, 3.7, 5.6, and 4.4 percentage points, respectively. These results support the complementary contributions of C3k2PMM, CGHFF, and C2MECM to classification and localization performance.

Among the formally published classroom-specific detectors, SLBDetection-Net provides the stronger comparison, reaching 73.6% Precision, 73.5% Recall, 79.4% mAP_50_, and 58.4% mAP_50:95_. MCHIME-Det improves these values by 2.7, 1.4, 2.7, and 3.0 percentage points, respectively. This pattern is consistent with recent classroom-specific studies showing that multi-scale feature preservation, selective attention, contextual interaction, and small-target processing are especially important in dense and occluded classroom scenes [7,8,9,12,13,16,22]. The proposed framework combines local–global spatial modeling, cross-level interaction, and memory-assisted semantic compensation while keeping model complexity close to the YOLO26n baseline.

For the qualitative visualizations, Average Confidence (AC) is the arithmetic mean of the confidence scores of all displayed predictions retained at the visualization threshold of 0.25. The calculation includes false-positive predictions and is performed directly on the retained detections without an additional ground-truth matching step. AC summarizes the mean confidence of the displayed detections, while detection accuracy is evaluated using the ground-truth-based mAP metrics in Table 6.

**Figure 7:**
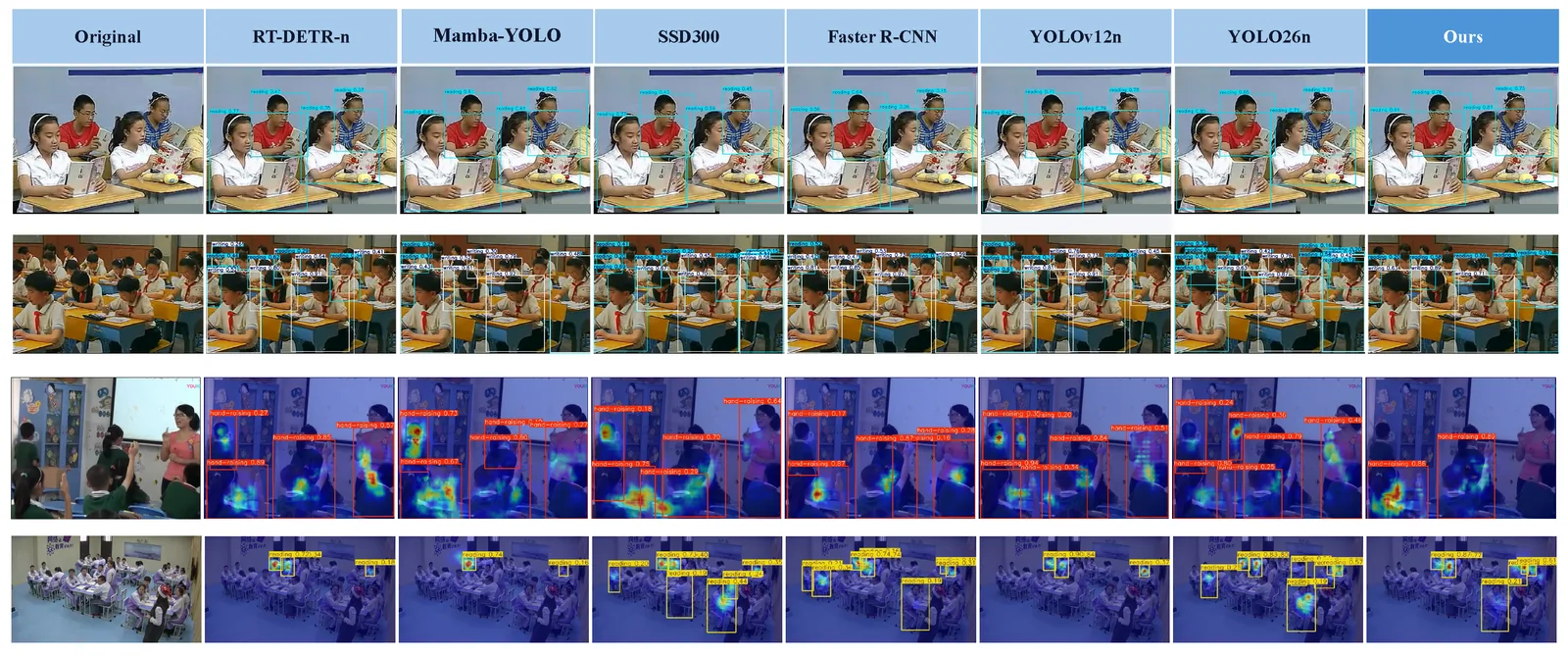
Qualitative comparison of representative detectors across four classroom scenes. The first column shows the original images, followed by the visualization results of RT-DETR-n, Mamba-YOLO-tiny, SSD300-MobileNetV2, Faster R-CNN, YOLOv12n, YOLO26n, and the proposed model.

Figure 7 presents qualitative results under different classroom layouts, viewpoints, and crowd densities. The visualizations show the predicted regions produced by each detector, while the ground-truth-based accuracy comparison is reported in Table 6. MCHIME-Det retains more complete responses around several small, crowded, and partially overlapping student regions in the illustrated scenes.

Overall, the quantitative and qualitative results demonstrate that MCHIME-Det is competitive with both general-purpose and classroom-specific detectors. The combination of C3k2PMM, CGHFF, and C2MECM improves feature discrimination, cross-level fusion, and localization in complex classroom scenes. Computational complexity and inference efficiency are further analyzed in Section 4.8.

### 4.3. Ablation Study

To systematically evaluate the effectiveness and complementarity of C3k2PMM, CGHFF, and C2MECM, a combinatorial ablation study was conducted. M0 denotes the original YOLO26n baseline; M1, M2, and M3 incorporate C3k2PMM, CGHFF, and C2MECM individually; M12, M13, and M23 represent their pairwise combinations; and M123 denotes the complete model. All variants were trained and evaluated under the same experimental protocol. Accuracy metrics are reported as mean ± standard deviation over five independent runs, whereas Params, GFLOPs, model size, and FPS are reported as single values.

As shown in Table 7, the three modules provide complementary improvements. Among the single-module variants, C3k2PMM (M1) increases Precision and mAP_50_ by 2.1 and 0.9 percentage points while reducing Params from 2.37 M to 2.11 M and GFLOPs from 5.2 G to 4.8 G. CGHFF (M2) provides the largest single-module Precision gain, reaching 73.6%, whereas C2MECM (M3) improves Recall to 73.4% and mAP_50:95_ to 58.9%.

The pairwise variants further confirm the complementarity of the three modules. M12 achieves 75.9% Precision and 80.1% mAP_50_, while M13 reaches 73.7% Recall and 60.1% mAP_50:95_ with only 2.13 M parameters. M23 further improves Recall and mAP_50:95_ to 74.3% and 60.6%, respectively.

With all three modules integrated, M123 achieves 76.3% Precision, 74.9% Recall, 82.1% mAP_50_, and 61.4% mAP_50:95_. Compared with M0, these correspond to improvements of 7.6, 3.7, 5.6, and 4.4 percentage points, respectively. Meanwhile, M123 retains 2.36 M parameters and 5.2 G FLOPs, essentially preserving the computational complexity of YOLO26n.

The internal operations also have distinct design roles. Within C3k2PMM, dilated depthwise groups address variation in the spatial extent of local cues, whereas directionally masked selective scanning provides broader contextual interaction. Within CGHFF, spatial recalibration suppresses irrelevant responses before reciprocal feature modulation, and grouped channel attention refines the fused representation. Within C2MECM, the input-dependent expert gate selects memory pathways, while global-pattern retrieval and spatial-context refinement provide complementary forms of compensation. The targeted comparisons in Table 8 further assess the contributions of the specified component configurations.

**Figure 8:**
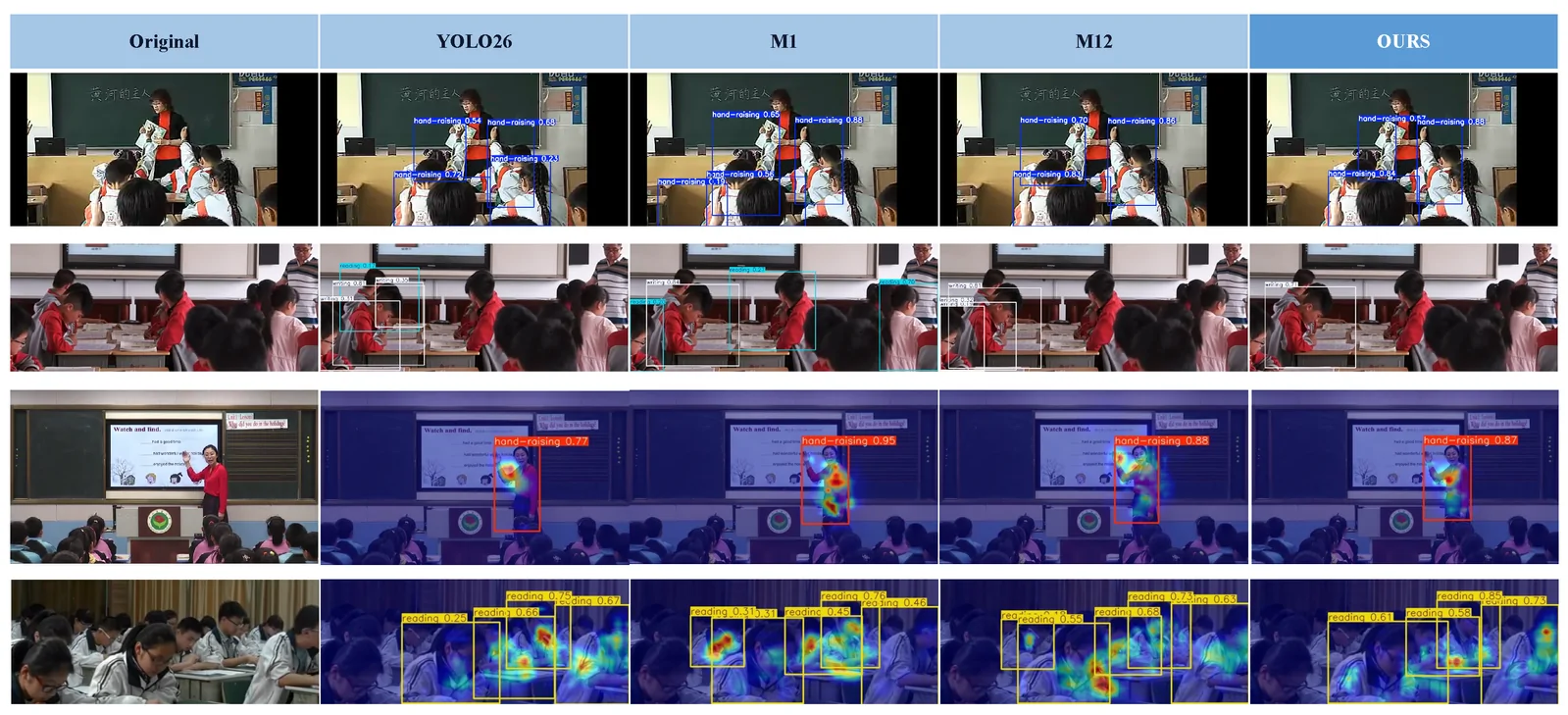
Qualitative visualization of representative ablation variants across four classroom scenes. The first column shows the original images, followed by the results of YOLO26n (M0), M1, M12, and the complete model (M123).

Figure 8 illustrates qualitative differences among the ablation variants. The complete model shows more complete and concentrated responses in several crowded and partially occluded examples. The corresponding detection-accuracy improvements are quantified by the mAP-based results in Table 7.

**Figure 9:**
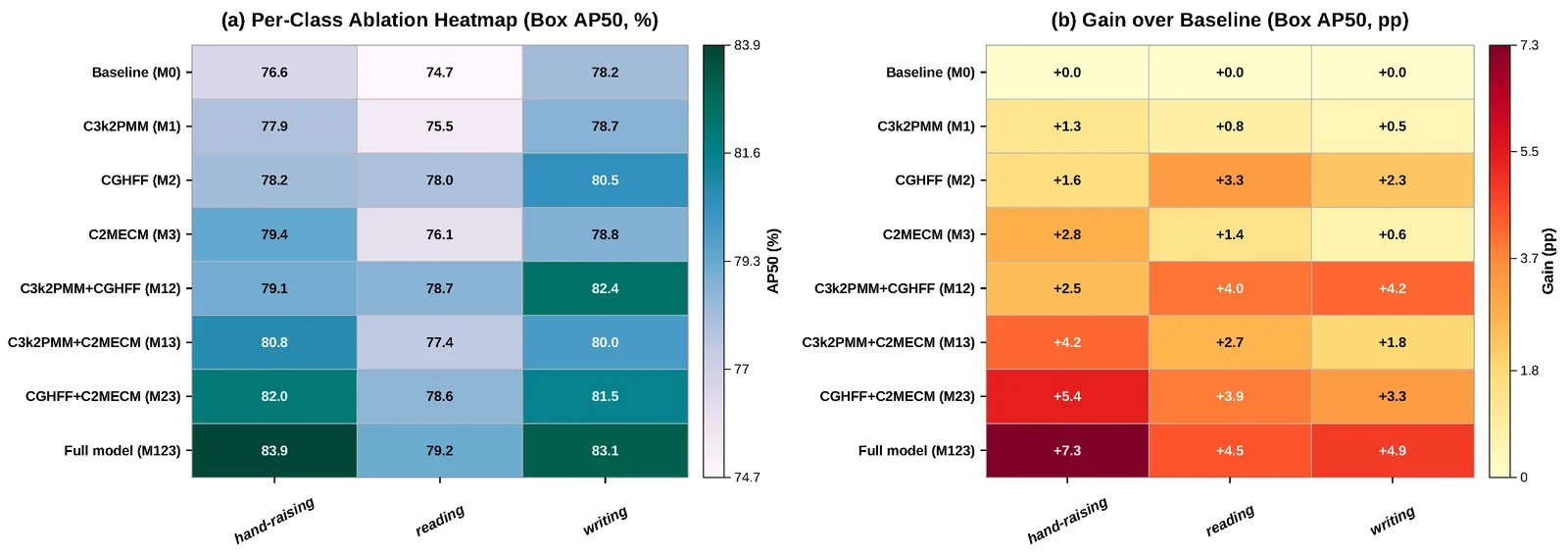
Per-class ablation analysis of the proposed modules. (**a**) Class-wise box AP_50_ values for hand-raising, reading, and writing. (**b**) Corresponding gains relative to the YOLO26n baseline in percentage points.

To further examine category-level behavior, Figure 9 reports the class-wise AP_50_ values and their gains relative to M0 for hand-raising, reading, and writing.

As shown in Figure 9, M123 improves all three behavior categories. Compared with M0, AP_50_ increases from 76.6% to 83.9% for hand-raising, from 74.7% to 79.2% for reading, and from 78.2% to 83.1% for writing, corresponding to gains of 7.3, 4.5, and 4.9 percentage points, respectively. The larger gain for hand-raising suggests that the proposed modules are particularly effective for behaviors involving localized and distinctive body structures, while the consistent gains for reading and writing demonstrate improved discrimination between visually similar activities.

Overall, the ablation results confirm that C3k2PMM, CGHFF, and C2MECM provide complementary improvements in feature representation, cross-level fusion, and semantic discrimination. Their integration improves both classification and localization performance while maintaining the lightweight characteristics of the original YOLO26n.

#### Internal Component Ablation

To further examine the contribution of the principal internal components, three targeted variants were constructed for C3k2PMM, and two variants were constructed for each of CGHFF and C2MECM. For C3k2PMM, M1-a removes the MGD-SS2D branch while retaining the multi-scale pyramid branch, whereas M1-b removes the multi-scale pyramid branch while retaining MGD-SS2D. M1-c retains both the multi-scale pyramid branch and standard SS2D scanning but removes directional masking, allowing the effect of directional masking to be evaluated separately. For CGHFF, M2-a removes Grouped Channel-Wise Self-Attention (GCSA), while M2-b removes reciprocal cross-level modulation. For C2MECM, M3-a removes the Spatial-Context Refinement Stream (SCStream), whereas M3-b replaces the multiple memory experts and sparse routing mechanism with a single memory expert. All variants were trained and evaluated using the same dataset partitions and experimental protocol as the main ablation study.

Each variant was run independently five times, and the corresponding mean and standard deviation are reported in Table 8.

Across five independent runs, the complete C3k2PMM configuration (M1) reaches 77.4±0.27% mAP_50_ and 57.8±0.34% mAP_50:95_. M1-a, which removes the MGD-SS2D branch, obtains 76.9±0.39% and 57.3±0.47%, while M1-b, which removes the multi-scale pyramid branch, obtains 76.7±0.46% and 57.6±0.32%. M1-c retains the multi-scale pyramid and standard SS2D scanning without directional masking and reaches 77.1±0.37% and 57.5±0.29%. The complete configuration therefore retains a 0.3-point mean advantage over M1-c in both metrics across the repeated runs.

For CGHFF, the complete configuration (M2) reaches 78.9±0.33% mAP_50_ and 58.5±0.41% mAP_50:95_. Removing GCSA (M2-a) reduces the repeated-run means by 0.7 and 0.6 points, whereas removing reciprocal cross-level modulation (M2-b) reduces them by 1.4 and 0.5 points. Reciprocal modulation therefore provides the larger contribution to the mean mAP_50_, while GCSA produces the slightly larger change in mean mAP_50:95_.

For C2MECM, the complete configuration (M3) reaches 78.1±0.36% mAP_50_ and 58.9±0.29% mAP_50:95_. Removing SCStream (M3-a) decreases the repeated-run means by 0.7 and 0.6 points. Replacing the multi-expert branch and sparse routing with a single expert (M3-b) yields 77.8±0.34% and 58.6±0.31%, corresponding to a 0.3-point mean difference in both metrics.

Across the three module groups, the complete configurations achieve the highest repeated-run means. The 0.3-point directional-masking and multi-expert mean differences are comparable to the observed run-to-run standard deviations and are therefore reported as modest component-level effects. The larger removals in the remaining comparisons identify the principal performance contributions inside each module.

### 4.4. Cross-Architecture Transferability

To examine whether the proposed modules are specifically dependent on YOLO26n, C3k2PMM, CGHFF, and C2MECM were further transferred to three representative lightweight YOLO architectures: YOLOv10n, YOLOv12n, and YOLOv13n. These models employ different feature-extraction and feature-fusion strategies while remaining within the lightweight one-stage detection paradigm.

The architecture-specific insertion positions were fixed before model training and evaluation according to each detector’s structural stages, including local feature extraction, P4/P3 cross-level fusion, and high-level semantic processing. In YOLOv10n, C3k2PMM replaced the C2f blocks at layers 2, 4, 6, 8, 13, 16, and 19; CGHFF replaced Concat layers 12 (P4) and 15 (P3); and C2MECM replaced the PSA block at layer 10, immediately after the SPPF block at layer 9. In YOLOv12n, C3k2PMM replaced the C3k2 blocks at layers 2, 4, and 20; CGHFF replaced Concat layers 10 (P4) and 13 (P3); and C2MECM was inserted after the A2C2f block at layer 8 and before the neck. In YOLOv13n, C3k2PMM replaced the DSC3k2 blocks at layers 2, 4, 17, 21, 26, and 30; CGHFF replaced Concat layers 16 (P4) and 20 (P3); and C2MECM was inserted after the A2C2f block at layer 8 and before the HyperACE block at layer 9. These mappings were specified before any test-set evaluation, and no insertion position was selected using test performance. The layer mappings listed above constitute the complete architecture-specific modifications. All unlisted layers, channel widths, strides, detection heads, and loss functions retain the corresponding base-model settings. The joint integration of all three components is denoted as “+All”. All variants were trained and evaluated using the same classroom dataset, input resolution, training configuration, and evaluation protocol as the main experiments. Parameter counts are also reported to distinguish performance gains from those obtained through model expansion. For repeatability analysis, the Baseline and “+All” configurations of each architecture were trained independently five times under the same fixed data partition and checkpoint-selection protocol; the three single-module variants remain point estimates.

As shown in Table 9, all three modules retain positive effects after being transferred to different lightweight YOLO architectures. C3k2PMM consistently improves detection accuracy while reducing the parameter count. CGHFF generally produces larger gains in mAP_50_, indicating that channel-guided hierarchical fusion remains effective under different neck structures. C2MECM also provides consistent improvements, with relatively stronger gains under the stricter mAP_50:95_ criterion, supporting the transferability of memory-assisted feature compensation. Across the five independent runs, the complete configuration improves mAP_50_ by 4.2, 5.1, and 3.8 percentage points on YOLOv10n, YOLOv12n, and YOLOv13n, respectively. The corresponding mAP_50:95_ gains are 3.1, 3.3, and 4.0 percentage points. The reported SDs for the repeated Baseline and “+All” configurations range from 0.28 to 0.48 percentage points.

**Figure 10:**
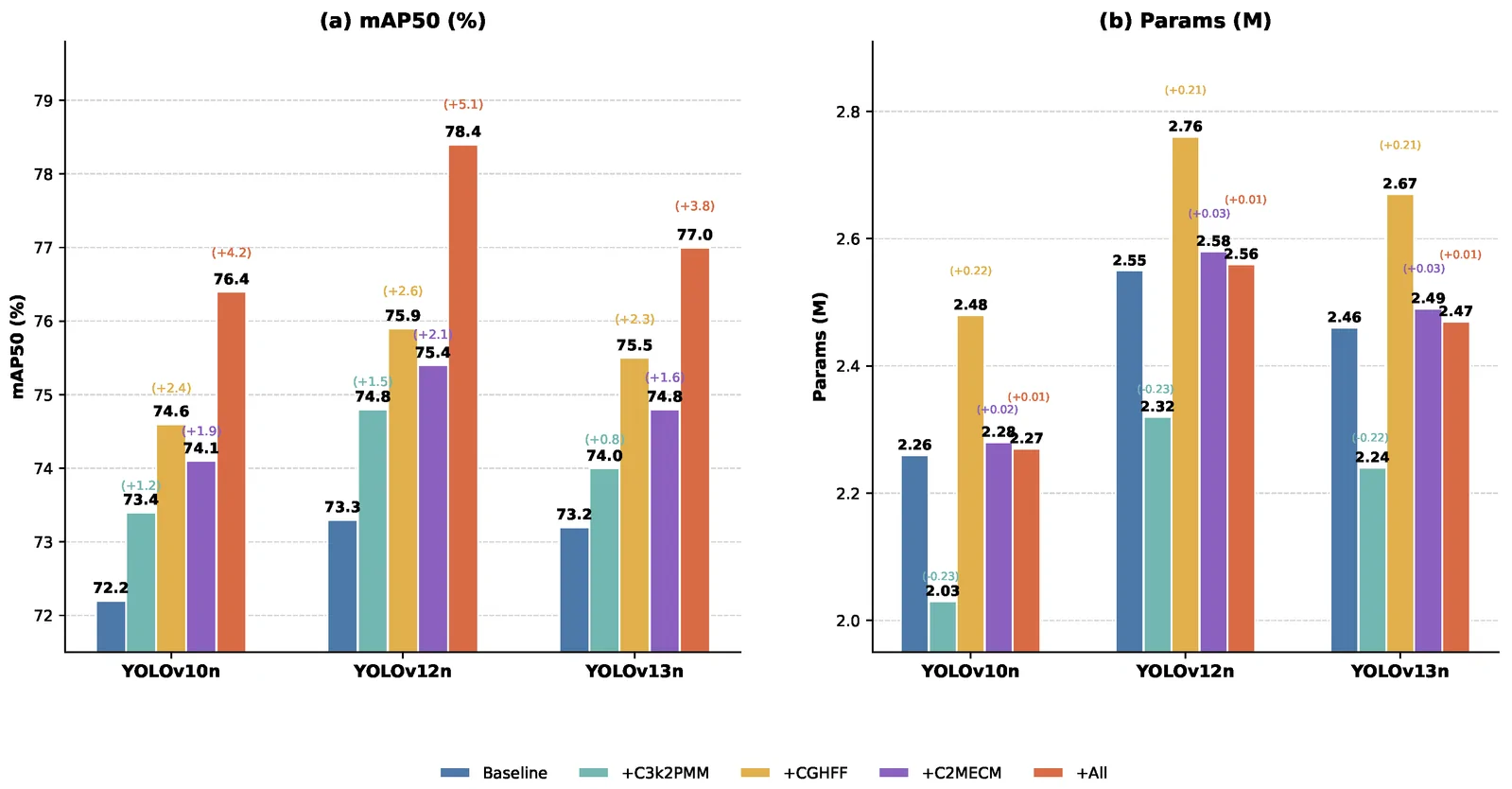
Cross-architecture transferability of the proposed modules. (**a**) Comparison of mAP_50_ across different lightweight YOLO architectures. Baseline and “+All” bars show the mean with SD error bars over five independent runs; the intermediate variants are point estimates. (**b**) Comparison of parameter counts for the corresponding model variants.

Figure 10 provides a direct visual comparison of performance and complexity across architectures. In Figure 10a, the Baseline and “+All” bars show the five-run means with SD error bars, whereas the three single-module variants are shown as point estimates. In Figure 10b, each “+All” variant adds only 0.01 M parameters relative to its corresponding baseline. The repeated Baseline–“+All” comparisons show that the complete module combination transfers across the three evaluated lightweight YOLO architectures without substantial model expansion.

### 4.5. Behavior-Wise Performance Analysis

To further examine the behavior-specific performance of MCHIME-Det, class-wise AP_50_ was compared with that of the original YOLO26n. The results are summarized in Table 10.

As shown in Table 10, MCHIME-Det improves AP_50_ across all three classroom behaviors. The largest improvement is observed for hand-raising, where AP_50_ increases from 76.6% to 83.9%, corresponding to a gain of 7.3 percentage points. This result is consistent with the design of C3k2PMM and CGHFF, which strengthen elongated body structures and cross-level spatial information that are particularly important for recognizing raised arms.

For reading and writing, AP_50_ improves by 4.5 and 4.9 percentage points, respectively. Reading remains the most challenging category, reaching 79.2% AP_50_, which is likely related to its visual similarity with writing and the limited differences in hand, head, and book-related regions. Nevertheless, the consistent improvements in both categories indicate that hierarchical feature fusion and memory-assisted compensation enhance discrimination between visually similar classroom activities.

Overall, the behavior-wise results show that MCHIME-Det improves all three target categories, with particularly pronounced gains for hand-raising, while reading–writing ambiguity remains the primary category-level challenge.

### 4.6. Classroom Scene Complexity Analysis

#### 4.6.1. Scale-Conditioned Performance

Student instances in classroom images exhibit substantial scale variation because of differences in camera distance and seating position. To examine the sensitivity of different detectors to target scale, an instance-level scale-conditioned evaluation was conducted on the test subset.

For the *i*-th ground-truth instance, the relative bounding-box area is defined as

(35) $$\rho_{i}=w_{i}h_{i}\times 100\%,$$

where $w_{i}$ and $h_{i}$ denote the normalized width and height of the corresponding bounding box.

To avoid defining scale intervals according to the test distribution, the thresholds were determined exclusively from the training set. The 25th, 50th, and 75th percentiles of $\rho_{i}$ were approximately 1.24%, 2.71%, and 5.93%, respectively. For clearer interpretation, these values were fixed at 1.2%, 2.7%, and 5.9% for all subsequent evaluations. Accordingly, student instances were divided into four groups: Small (ρ<1.2%), Medium (1.2%≤ρ<2.7%), Large (2.7%≤ρ<5.9%), and Very Large (ρ≥5.9%).

As shown in Table 11, the four scale groups are relatively balanced in the test set, containing 1776, 1878, 1787, and 1755 instances, respectively. This distribution provides sufficient samples for evaluating scale-dependent detection behavior without introducing severe group imbalance.

Predictions were generated only once on the complete test set, and scale-conditioned AP_50_ was subsequently calculated using a group-specific ignore protocol. For a target scale group *g*, ground-truth instances within *g* were treated as evaluable objects, whereas instances belonging to other scale groups were retained in the image but marked as ignored. Predictions were matched class-wise at IoU = 0.50 using standard one-to-one assignment, with evaluable ground truths taking priority over ignored instances.

For an unmatched prediction ${\hat{B}}_{k}$, its relative area was defined as

(36) $${\hat{\rho }}_{k}={\hat{w}}_{k}{\hat{h}}_{k}\times 100\%,$$

where ${\hat{w}}_{k}$ and ${\hat{h}}_{k}$ denote the normalized width and height of the predicted bounding box. An unmatched prediction was counted as a false positive only when ${\hat{\rho }}_{k}$ fell within the scale interval currently being evaluated; otherwise, it was ignored. Unmatched in-group ground-truth instances were counted as false negatives. After applying the group-specific ignore masks, class-wise AP_50_ was computed and averaged across the three behavior categories.

A fixed subset of representative detectors was used for the scale-, density-, and overlap-conditioned analyses: RT-DETR-n, SSD300-MobileNetV2, Mamba-YOLO-tiny, YOLO-Worldv2-n, YOLOv12n, and the direct YOLO26n baseline. This subset covers different architectural families while avoiding redundant repetition of closely related YOLO variants, and the same comparator set is retained across all three conditional analyses. The results are reported in Table 12.

Scale Gap is defined as

(37) $$\mathrm{Scale}\;\mathrm{Gap}=AP_{50}^{\mathrm{Very}\;\mathrm{Large}}-AP_{50}^{\mathrm{Small}},$$

where a smaller value indicates lower sensitivity to target-scale variation. Small Gain is defined relative to YOLO26n as

(38) $$\mathrm{Small}\;\mathrm{Gain}=AP_{50,\mathrm{model}}^{\mathrm{Small}}-AP_{50,\mathrm{YOLO}26n}^{\mathrm{Small}}.$$

As shown in Table 12, detection performance generally improves with increasing student scale, confirming that limited spatial evidence remains a major difficulty in classroom behavior detection. MCHIME-Det achieves the highest mAP_50_ across all four scale groups, reaching 75.4%, 80.7%, 84.0%, and 85.8% from Small to Very Large targets, respectively.

The advantage is most pronounced for Small instances. MCHIME-Det exceeds YOLO26n by 8.3 percentage points in this group, which is larger than its overall mAP_50_ gain of 5.6 points. This result indicates that the combination of multi-scale local modeling, cross-level feature fusion, and memory-assisted compensation is particularly beneficial when student behavior cues occupy limited image regions.

MCHIME-Det also exhibits the smallest Scale Gap of 10.4 percentage points, compared with 14.2 points for YOLO26n and 12.9 points for YOLO-Worldv2-n. These results indicate that the proposed framework not only improves overall detection accuracy but also reduces sensitivity to student scale variation, with the largest benefit observed for small and distant classroom targets.

The stronger gain on small instances is consistent with the broader object-detection literature, where scale variation and insufficient spatial evidence have long been identified as major sources of performance degradation [35,56]. Related fine-grained detection studies have further shown that super-resolution-enhanced YOLO frameworks can improve recognition when target appearance contains limited spatial detail [57]. Similar difficulties have been reported in classroom-specific detectors for distant, small, and densely distributed students [7,14]. The present results extend that observation by showing that the benefit of MCHIME-Det is disproportionately large in the Small subset rather than being uniform across target scales.

#### 4.6.2. Density-Conditioned Performance

Classroom scenes contain substantially different numbers of visible students, ranging from sparse scenes with only a few annotated instances to densely occupied classrooms. Increasing student density generally introduces more inter-instance interference, partial occlusion, and background complexity. To evaluate detector robustness under different classroom densities, an image-level density-conditioned analysis was conducted.

For the *j*-th image, classroom density is defined as the number of annotated student behavior instances:

(39) $$D_{j}=N_{j},$$

where $N_{j}$ denotes the number of ground-truth bounding boxes in image *j*.

As with the scale-conditioned analysis, the grouping thresholds were determined exclusively from the training set. The 33rd and 67th percentiles of the image-level instance-count distribution were 2 and 6, respectively. Accordingly, classroom images were divided into three groups: Low (D≤2), Medium (3≤D≤6), and High (D≥7).

As shown in Table 13, the three density subsets contain 446, 416, and 327 test images, respectively. Although the number of high-density images is smaller, each subset contains sufficient samples for evaluating performance under progressively more crowded classroom conditions.

Unlike the instance-level scale analysis, density is defined at the image level. Therefore, the test subset was directly partitioned into three image subsets according to $D_{j}$, and each detector was evaluated separately on the Low-, Medium-, and High-density subsets using the same mAP_50_ protocol as the overall evaluation.

Density Drop is defined as

(40) $$\mathrm{Density}\;\mathrm{Drop}=AP_{50}^{\mathrm{Low}}-AP_{50}^{\mathrm{High}},$$

where a smaller value indicates greater robustness to increasing classroom density. High Gain is defined relative to YOLO26n as

(41) $$\mathrm{High}\;\mathrm{Gain}=AP_{50,\mathrm{model}}^{\mathrm{High}}-AP_{50,\mathrm{YOLO}26n}^{\mathrm{High}}.$$

As shown in Table 14, detection performance generally decreases as classroom density increases. This trend indicates that crowded scenes introduce additional difficulty through stronger inter-student interference and partial occlusion.

MCHIME-Det achieves 84.8%, 82.4%, and 78.0% mAP_50_ on the Low-, Medium-, and High-density subsets, respectively, consistently outperforming the comparison models. The advantage is most evident in High-density scenes, where MCHIME-Det exceeds YOLO26n by 7.2 percentage points.

Moreover, MCHIME-Det exhibits the smallest Density Drop of 6.8 percentage points, compared with 9.7 points for YOLO26n and 8.9 points for YOLO-Worldv2-n. These results indicate that the proposed feature extraction, hierarchical fusion, and memory compensation mechanisms improve robustness under increasing classroom crowding, particularly when multiple student instances interact within the same scene.

This monotonic degradation with increasing density also agrees with crowded-object detection studies, where nearby instances and occlusion can increase localization ambiguity [58]. Classroom-specific work likewise identifies dense student distributions as a major source of missed and confused detections [12,13]. The smaller Density Drop of MCHIME-Det therefore provides evidence that the proposed hierarchical representation is more resilient to inter-student interference as scene occupancy increases.

#### 4.6.3. Overlap-Conditioned Performance

In classroom scenes, neighboring students frequently exhibit substantial spatial overlap because of dense seating, body pose, and partial occlusion. Such conditions increase ambiguity in instance boundaries and reduce the visible behavioral evidence available for detection. To evaluate the sensitivity of different detectors to inter-instance spatial interference, an instance-level overlap-conditioned analysis was conducted on the test subset.

The dataset provides bounding-box annotations rather than visible-region masks or depth-order information. The overlap metric therefore measures geometric bounding-box overlap, whereas physical occlusion refers to the fraction of student evidence that remains visible.

For two ground-truth instances $B_{i}$ and $B_{j}$ in the same image, their normalized overlap ratio is defined as

(42) $$O_{ij}=\frac{|B_{i}\cap B_{j}|}{\min(|B_{i}|,|B_{j}|)},$$

where $|B_{i}\cap B_{j}|$ denotes the intersection area between the two bounding boxes, while $|B_{i}|$ and $|B_{j}|$ denote their respective areas. Compared with conventional IoU, this definition is more sensitive when a relatively small student box is largely contained within a larger neighboring box.

The instance-level overlap score is then defined as

(43) $$O_{i}=\max_{j\neq i}O_{ij},$$

with $O_{i}=0$ when no other annotated student is present in the same image.

To avoid selecting thresholds according to the test distribution, the overlap intervals were determined exclusively from the training set. Based on the training-set overlap distribution, the thresholds were fixed at 0.05, 0.30, and 0.50. Accordingly, student instances were divided into four groups: Low (O<0.05), Mild (0.05≤O<0.30), Moderate (0.30≤O<0.50), and Heavy (O≥0.50).

Table 15 summarizes these thresholds and the split-specific instance counts for the four overlap groups.

The four overlap groups are relatively balanced in the test set, containing 1762, 1751, 1894, and 1789 instances, respectively. This distribution provides sufficient samples for evaluating performance under progressively stronger spatial interference.

As in the scale-conditioned evaluation, predictions were generated only once on the complete test set. For a target overlap group *g*, ground-truth instances belonging to *g* were treated as evaluable objects, whereas instances from other overlap groups were retained in the image but marked as ignored. Predictions were matched class-wise at IoU = 0.50 using one-to-one assignment, with evaluable ground truths taking priority over ignored instances.

For unmatched predictions, an overlap score was calculated using the same geometric definition. For a predicted box ${\hat{B}}_{k}$,

(44) $${\hat{O}}_{k}=\max_{\ell \neq k}\frac{|{\hat{B}}_{k}\cap {\hat{B}}_{\ell }|}{\min(|{\hat{B}}_{k}|,|{\hat{B}}_{\ell }|)},$$

with ${\hat{O}}_{k}=0$ when no other candidate prediction is present in the image. An unmatched prediction was counted as a false positive only when its prediction-side overlap score fell within the overlap interval currently being evaluated; otherwise, it was ignored. Unmatched in-group ground-truth instances were counted as false negatives.

After applying the group-specific ignore masks, class-wise AP_50_ was computed and averaged across the three classroom behavior categories. The results are summarized in Table 16.

The common scale-, density-, and overlap-conditioned evaluation procedure, including prediction post-processing, duplicate-prediction handling, the ignore protocol, and AP integration, is summarized in Algorithm 2.
**Algorithm 2** Conditioned AP_50_ evaluation for scale, density, and overlap groups**Input:** Test images D, predictions P, ground truths G, group *g*, matching IoU τ=0.50**Output:** Conditioned ${\mathrm{mAP}}_{50}^{g}$ 1: Generate predictions once on the full test set and apply each detector’s native post-processing once 2: **if** detector uses NMS **then** 3:     Apply class-aware NMS ($\tau_{\mathrm{NMS}}=0.70$, $\tau_{\mathrm{conf}}=0.001$, max_det=300) 4: **else** 5:     Retain the decoded native Top-*K* predictions without additional NMS 6: **end if** 7: **if**
*g* is an image-density group **then** 8:     $D_{g}\leftarrow \left\{I\in D:\right|G_{I}\left|\;\mathrm{lies}\;\mathrm{in}\;\mathrm{density}\;\mathrm{group}\;g\right\}$ 9:     **return** standard ${\mathrm{mAP}}_{50}$ evaluated on $D_{g}$ without ignore masks10: **end if**11: **for all** classes c∈C **do**12:     $R_{c}\leftarrow \emptyset$; $N_{c}^{+}\leftarrow 0$▹ TP/FP records; GT count13:     **for all** images I∈D **do**14:         $E\leftarrow \{b\in G_{I,c}:b\in g\}$; $Q\leftarrow G_{I,c}\setminus E$15:         Mark E,Q as unmatched; $N_{c}^{+}\leftarrow N_{c}^{+}+\left|E\right|$16:         **for all** class-*c* predictions *p* in *I*, sorted by descending confidence **do**17:            $e^{*}\leftarrow \arg\max_{e\in E_{\mathrm{unmatched}}}\mathrm{IoU}\left(p,e\right)$ (if nonempty)18:            **if** $e^{*}$ exists and $\mathrm{IoU}(p,e^{*})\geq \tau$ **then**19:                Mark $e^{*}$ as matched; append $\left(s_{p},1,0\right)$ to $R_{c}$▹ TP20:            **else**21:                $d\leftarrow \max_{e\in E_{\mathrm{matched}}}\mathrm{IoU}\left(p,e\right)$ (or 0 if empty)22:                **if** d≥τ **then**23:                    **if** PredInGroup(p,g) **then**24:                        Append $\left(s_{p},0,1\right)$ to $R_{c}$▹ Duplicate FP25:                    **end if**26:                **else**27:                    $q^{*}\leftarrow \arg\max_{q\in Q_{\mathrm{unmatched}}}\mathrm{IoU}\left(p,q\right)$ (if nonempty)28:                    **if** $q^{*}$ exists and $\mathrm{IoU}(p,q^{*})\geq \tau$ **then**29:                        Mark $q^{*}$ as matched; ignore *p*30:                    **else if** PredInGroup(p,g) **then**31:                        Append $\left(s_{p},0,1\right)$ to $R_{c}$▹ Unmatched FP32:                    **end if**33:                **end if**34:            **end if**35:         **end for**36:     **end for**37:     Sort $R_{c}$ by confidence and compute cumulative ${\mathrm{TP}}_{c}\left(k\right),{\mathrm{FP}}_{c}\left(k\right)$38:     $P_{c}\left(k\right)\leftarrow \frac{{\mathrm{TP}}_{c}\left(k\right)}{{\mathrm{TP}}_{c}\left(k\right)+{\mathrm{FP}}_{c}\left(k\right)}$; $R_{c}\left(k\right)\leftarrow \frac{{\mathrm{TP}}_{c}\left(k\right)}{N_{c}^{+}}$39:     $P_{c}^{\mathrm{interp}}\left(r\right)\leftarrow \max_{\tilde{r}\geq r}P_{c}\left(\tilde{r}\right)$40:     ${\mathrm{AP}}_{50}^{c,g}\leftarrow \frac{1}{101}\sum_{r\in \{0,0.01,\ldots ,1\}}P_{c}^{\mathrm{interp}}\left(r\right)$41: **end for**42: ${\mathrm{mAP}}_{50}^{g}\leftarrow \frac{1}{|C_{g}|}\sum_{c\in C_{g}}{\mathrm{AP}}_{50}^{c,g}$43: **return** 
${\mathrm{mAP}}_{50}^{g}$

For scale-conditioned evaluation, PredInGroup(p,g) checks whether the relative predicted-box area defined in Equation (36) belongs to *g*; for overlap-conditioned evaluation, it uses the predicted-box overlap in Equation (44), computed from the fixed post-processed prediction set. Density groups are evaluated as image subsets with ordinary AP_50_ and without instance-level ignore masks. The mean is computed over behavior classes having evaluable ground-truth instances.

Overlap Drop is defined as

(45) $$\mathrm{Overlap}\;\mathrm{Drop}=AP_{50}^{\mathrm{Low}}-AP_{50}^{\mathrm{Heavy}},$$

where a smaller value indicates greater robustness to increasing spatial overlap. Heavy Gain is defined relative to YOLO26n as

(46) $$\mathrm{Heavy}\;\mathrm{Gain}=AP_{50,\mathrm{model}}^{\mathrm{Heavy}}-AP_{50,\mathrm{YOLO}26n}^{\mathrm{Heavy}}.$$

As shown in Table 16, detection accuracy generally decreases as spatial overlap increases, confirming that inter-student interference remains an important source of difficulty in classroom behavior detection. MCHIME-Det consistently achieves the highest mAP_50_ across all four overlap levels, reaching 85.5%, 83.0%, 80.4%, and 76.9% for the Low, Mild, Moderate, and Heavy subsets, respectively.

The advantage becomes more pronounced under Heavy overlap, where the proposed model exceeds YOLO26n by 7.0 percentage points. In addition, its Overlap Drop is limited to 8.6 points, compared with 11.8 points for YOLO26n and 10.4 points for YOLO-Worldv2-n.

These results indicate that the proposed framework better preserves behavior-discriminative information as inter-instance overlap increases. The combination of local–global spatial modeling, selective cross-level fusion, and memory-assisted compensation is particularly beneficial when student targets partially interfere with one another in crowded classroom scenes.

The observed trend is consistent with prior crowded-object research showing that spatial overlap and occlusion can degrade localization when neighboring instances provide competing visual evidence [58]. The issue is especially relevant to classrooms because adjacent students may share similar poses and weak local boundaries [12,14]. The reduced Overlap Drop therefore complements the overall mAP results by quantifying how rapidly performance deteriorates as geometric interference becomes stronger.

### 4.7. Cross-Source Evaluation

Evaluation on an image-level split of pooled sources may not capture performance degradation caused by real-world distribution shifts. Differences in camera viewpoint, classroom layout, student density, image acquisition, and annotation practice can change the target distribution, a broader problem emphasized in out-of-distribution and domain-generalization benchmarks [59,60]. To examine detector performance on separately published data sources, cross-source experiments were conducted on three external classroom behavior datasets: STBD-08, Student Behaviour Detection (SBD), and Student Classroom Behavior Dataset (SCBD).

The publicly available STBD-08 dataset [14] used in this study contains 8884 images, including 7052 training and 1832 validation images. SBD [61] contains 2471 classroom images. SCBD [62] contains 2032 images. The SCB-05 three-class subset corresponds to the same 6864-image collection as SCB-2025-7-public-1, which was used for model development. Accordingly, all 6864 SCB-05 images were excluded from the external evaluation because they belong to the model-development collection. The 6864-image count is a dataset-source exclusion count. Exact and visually near-duplicate overlap was checked separately for the three retained external sources.

All external datasets were standardized to the same three behavior categories: hand-raising, reading, and writing. Categories outside this taxonomy were removed, and minor naming differences such as read and write were standardized without semantic category merging. For the three retained external sources (STBD-08, SBD, and SCBD), hash-based image comparison and manual visual inspection were applied before final sample selection. The recorded pre-sampling removal counts (exact, confirmed visually near-duplicate) were (0,0) for STBD-08, (0,0) for SBD, and (0,0) for SCBD. The final fixed 1000-image subsets also contained zero confirmed exact or visually near-duplicate images overlapping with the model-development corpus. The verified scope is image-level overlap; recording, camera, and classroom identifiers are unavailable for group-level independence analysis. To provide an identical evaluation budget, 1000 images were sampled without replacement from each retained source after category harmonization and duplicate screening, using a fixed random seed of 42. The source-provided image IDs were retained to define the selected subset, which was fixed before evaluation and reused unchanged for every detector in both cross-source settings and across all repeated runs. The resulting class-specific instance counts are reported in Table 17.

All annotations were converted to the same detection format used in the main experiments. Two complementary settings were adopted: zero-shot evaluation and dataset-specific retraining.

#### 4.7.1. Zero-Shot Cross-Source Evaluation

In the zero-shot setting, all detectors were trained only on the two-source public dataset and directly evaluated on the complete 1000-image subset of each external source. No target-domain images were used for fine-tuning, parameter updating, checkpoint selection, or threshold recalibration.

As shown in Table 18, performance varies considerably across external sources, showing source-dependent performance variation under the evaluated protocol. MCHIME-Det achieves the highest mAP_50_ on all three retained datasets, ranging from 67.6% on SCBD to 72.2% on STBD-08. Its mean mAP_50_ reaches 69.7%, exceeding the stronger mean comparator (Best Other at 66.5%) by 3.2 percentage points. Relative to the stronger comparator on each dataset, the gains range from 2.7 to 3.3 points, indicating higher zero-shot detection accuracy across these three sampled external sources under the reported protocol.

#### 4.7.2. Cross-Source Retraining Evaluation

To further evaluate target-domain adaptability, each fixed 1000-image subset was divided once, using the same random seed of 42, into 700 training images, 100 validation images, and 200 held-out test images. The source image IDs assigned to these three subsets were fixed before training and reused for every detector and all five independent runs. The training subset was used for parameter updating, the validation subset for checkpoint selection, and the test subset only for final evaluation. Recording and classroom identifiers are unavailable, so the protocol evaluates held-out images from each sampled source rather than group-disjoint recordings or classrooms.

This experiment characterizes held-out-image performance within each sampled source after target-source supervision. The zero-shot evaluation in Section 4.7.1 provides the primary evidence for cross-source transfer.

Table 19 reports the five-run retraining results under this fixed partition.

After dataset-specific retraining, all detectors show substantial performance improvements. MCHIME-Det remains the strongest across all three retained external datasets, achieving mAP_50_ values of 82.6%, 81.0%, and 79.6%, respectively. Its mean mAP_50_ reaches 81.1%, exceeding the stronger mean comparator (Best Other at 78.5%) by 2.5 percentage points.

The smaller margin after retraining compared with zero-shot evaluation is expected because all models receive target-domain supervision. The retraining results therefore quantify source-specific adaptation on held-out images within each sampled source and are interpreted separately from the zero-shot cross-source transfer results.

### 4.8. Accuracy–Efficiency Trade-Off

To further evaluate the balance between detection accuracy and computational efficiency, representative general-purpose and classroom-specific detectors were compared in terms of parameter count, computational complexity, model size, and inference speed. The corresponding detection accuracy is reported in Table 6. Table 20 summarizes the associated model-complexity and forward-pass efficiency measurements.

Compared with the YOLO26n baseline, MCHIME-Det reduces the parameter count slightly from 2.37 M to 2.36 M while retaining the same computational cost of 5.2 GFLOPs and the same reported disk file size of 5.1 MB. MCHIME-Det was stored as an FP16 checkpoint (5.1 MB) but benchmarked in FP32. In contrast, SSD300-MobileNetV2 used FP32 for both checkpoint storage and inference and operated at 300-by-300 input resolution; other detectors were tested at 640-by-640. Its GFLOPs and FPS must therefore be interpreted under a different image-resolution setting. Meanwhile, mAP_50_ and mAP_50:95_ increase by 5.6 and 4.4 percentage points, respectively, with only a modest decrease in inference speed from 190 to 182 FPS.

**Figure 11:**
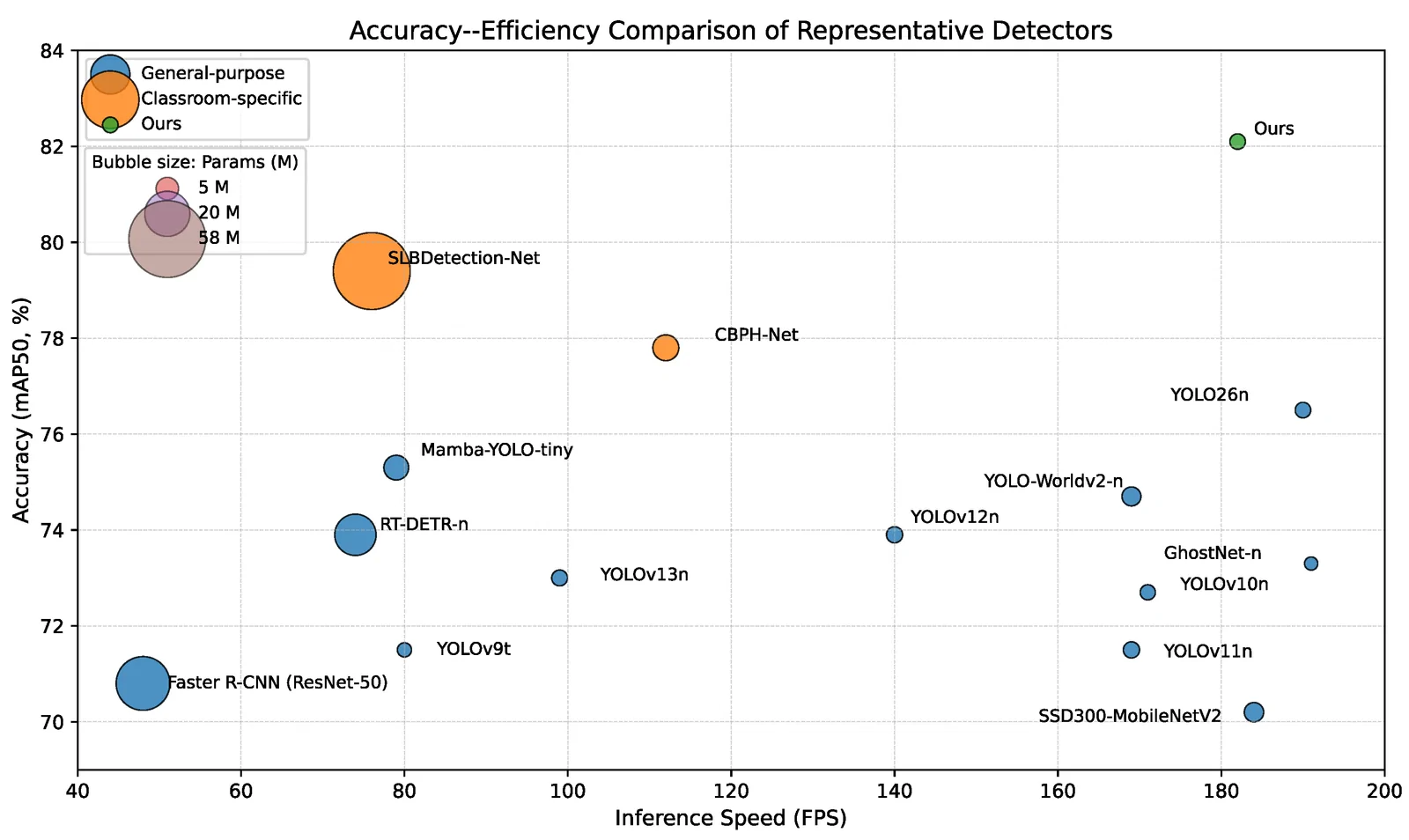
Accuracy–efficiency comparison of representative detectors. The horizontal axis denotes FPS, the vertical axis denotes mAP_50_, and bubble size represents the number of parameters.

As shown in Figure 11, several lightweight detectors achieve high inference throughput but provide lower detection accuracy. Among the classroom-specific methods, CBPH-Net achieves stronger task-specific detection performance than many general-purpose detectors, but requires 6.58 M parameters and 16.10 GFLOPs while operating at 112 FPS. SLBDetection-Net (Swin) is considerably heavier, with 58.00 M parameters and 208.9 GFLOPs, and reaches only 76 FPS. In comparison, MCHIME-Det achieves 82.1% mAP_50_ with only 2.36 M parameters and 5.2 GFLOPs while maintaining 182 FPS.

The proposed C3k2PMM, CGHFF, and C2MECM modules improve feature representation while preserving the lightweight characteristics of the YOLO26n baseline. MCHIME-Det therefore provides a favorable accuracy–efficiency trade-off, combining high detection accuracy, low computational complexity, and high model-level forward-pass throughput on the RTX 4090.

### 4.9. Cross-Hardware Inference Analysis

**Figure 12:**
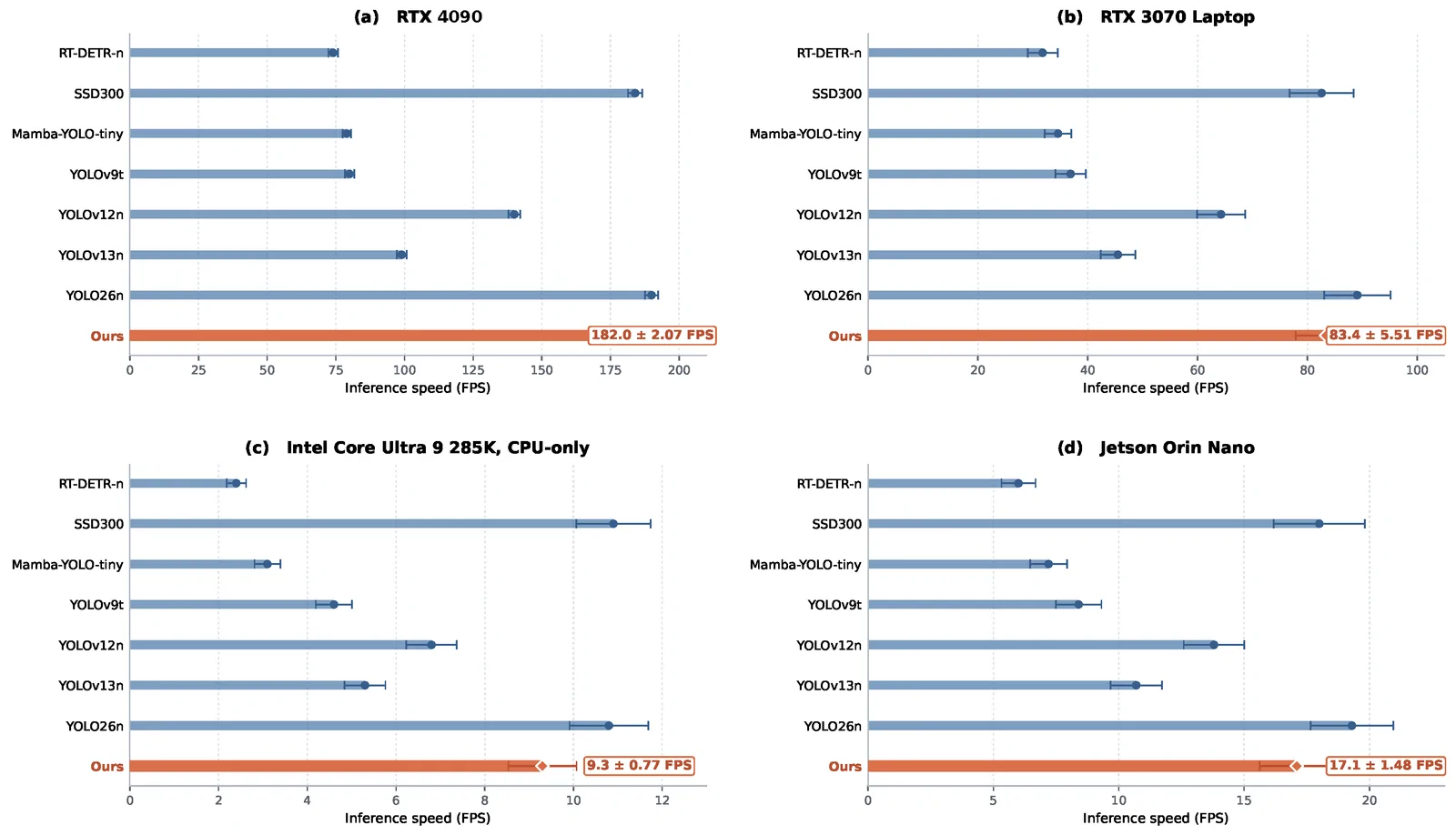
Cross-hardware inference-speed comparison of representative detectors on four computing platforms: (**a**) RTX 4090, (**b**) RTX 3070 Laptop, (**c**) Intel Core Ultra 9 285K CPU-only, and (**d**) Jetson Orin Nano. Error bars denote the standard deviation over repeated measurements. Numerical values are displayed only for MCHIME-Det to reduce visual congestion.

To characterize model-level inference behavior beyond a single desktop GPU, the forward-pass performance of representative detectors was examined across four hardware platforms: an NVIDIA RTX 4090 desktop GPU, an RTX 3070 Laptop GPU, an Intel Core Ultra 9 285K CPU-only platform, and a Jetson Orin Nano edge device. Models were evaluated at an input resolution of 640×640 with a batch size of 1 except SSD300-MobileNetV2, which used a 300-by-300 input. Repeated measurements were performed to account for runtime variation, and the results are shown in Figure 12.

As shown in Figure 12, MCHIME-Det maintains high inference throughput across heterogeneous hardware platforms, reaching 182.0±2.07 FPS on the RTX 4090 and 83.4±5.51 FPS on the RTX 3070 Laptop GPU. Under more resource-constrained conditions, it achieves 9.3±0.77 FPS on the CPU-only platform and 17.1±1.48 FPS on the Jetson Orin Nano.

The Jetson Orin Nano 8 GB Developer Kit ran Ubuntu 20.04 with JetPack 5.1.2 (L4T 35.4.1), CUDA 11.4.19, cuDNN 8.6.0, Python 3.10, and PyTorch 2.1.0. Model execution used FP32 precision with the PyTorch CUDA eager backend in 15 W power mode with locked device clocks. Model fusion was enabled and gradients were disabled. The benchmark used 640-by-640 inputs, a batch size of 1, 200 warm-up passes, and 1000 timed forward passes. The timing scope covers the network forward pass; preprocessing, data transfer, and post-processing are outside the reported FPS.

Compared with the original YOLO26n, MCHIME-Det retains approximately 95.8%, 93.6%, 86.1%, and 88.6% of the baseline inference throughput on the RTX 4090, RTX 3070 Laptop, CPU, and Jetson platforms, respectively. The larger relative speed reduction on CPU and edge hardware indicates that the additional multi-branch, selective-scan, and memory-assisted operations are more sensitive to operator implementation and memory-access efficiency under constrained computing resources [63].

MCHIME-Det remains faster than the heavier RT-DETR-n and Mamba-YOLO-tiny architectures across all four tested platforms. On the Jetson Orin Nano, the model-level benchmark reaches 17.1 FPS, a rate relevant to reduced-rate classroom video sampling on the tested device [64]. The feasibility of combining deep-learning-based visual perception with mobile sensing platforms has likewise been demonstrated in cost-effective ecological monitoring using amphibious unmanned aerial vehicles [65]. Relative to YOLO26n, the additional feature-processing operations reduce forward-pass throughput while increasing mAP_50_ and mAP_50:95_ by 5.6 and 4.4 percentage points, respectively.

Overall, the cross-hardware results demonstrate model-level inference portability under the four tested computing environments. The two GPU platforms provide high-rate inference, whereas the CPU-only and Jetson results represent lower-rate processing under more constrained hardware.

The Jetson Orin Nano result of 17.1 FPS corresponds to approximately 58.5 ms per model inference under the reported 640-by-640, batch-size-one benchmark. This rate supports periodic or reduced-rate sampling of a single classroom video stream for behavior summaries. Continuous 25- or 30-FPS processing requires higher throughput, and complete system latency additionally includes video decoding, preprocessing, data transfer, and post-processing.

### 4.10. Robustness Analysis

Robustness under common image corruptions is a standard way to probe whether visual models retain performance when the test distribution is degraded [66,67,68]. Related degradation-aware object detection studies have also investigated challenging image-quality conditions [69]. Following this general evaluation paradigm, five perturbations relevant to classroom monitoring were considered: brightness variation, Gaussian noise, motion blur, JPEG compression, and resolution degradation. In particular, illumination variation can substantially alter local appearance and boundary contrast, and explicit illumination adaptation has also been investigated in challenging visual environments such as underwater coral segmentation [70]. The severity values are specific to the present classroom evaluation and differ from the ImageNet-C severity scale. All models were trained only on the clean training set, while perturbations were introduced at the evaluation stage without additional fine-tuning. RT-DETR-n, Mamba-YOLO-tiny, YOLOv12n, YOLO26n, and the proposed model were selected for comparison. The perturbation settings are summarized in Table 21.

Each corruption setting was generated once from the same clean evaluation images, and the resulting corrupted image set was fixed and reused unchanged by all models. Ground-truth annotations and evaluation parameters remained unchanged. Brightness adjustment multiplied all pixel intensities by the specified factor and clipped the result to the valid 8-bit range. Gaussian noise used fixed random seed 42 and was sampled independently for every pixel and color channel in the 0–255 intensity domain, with zero mean and the standard deviation listed in Table 21; the perturbed values were clipped to [0,255] and converted back to the original image type.

Motion blur used a horizontal normalized linear kernel: for a kernel size *k*, the central row contained 1/k and all remaining entries were zero, while k=0 denoted the clean image. JPEG compression consisted of one encoding–decoding cycle at the reported quality factor. Resolution degradation first reduced both spatial dimensions by the specified scale factor using bilinear interpolation and then restored the image to the detector input resolution, again using bilinear interpolation.

**Figure 13:**
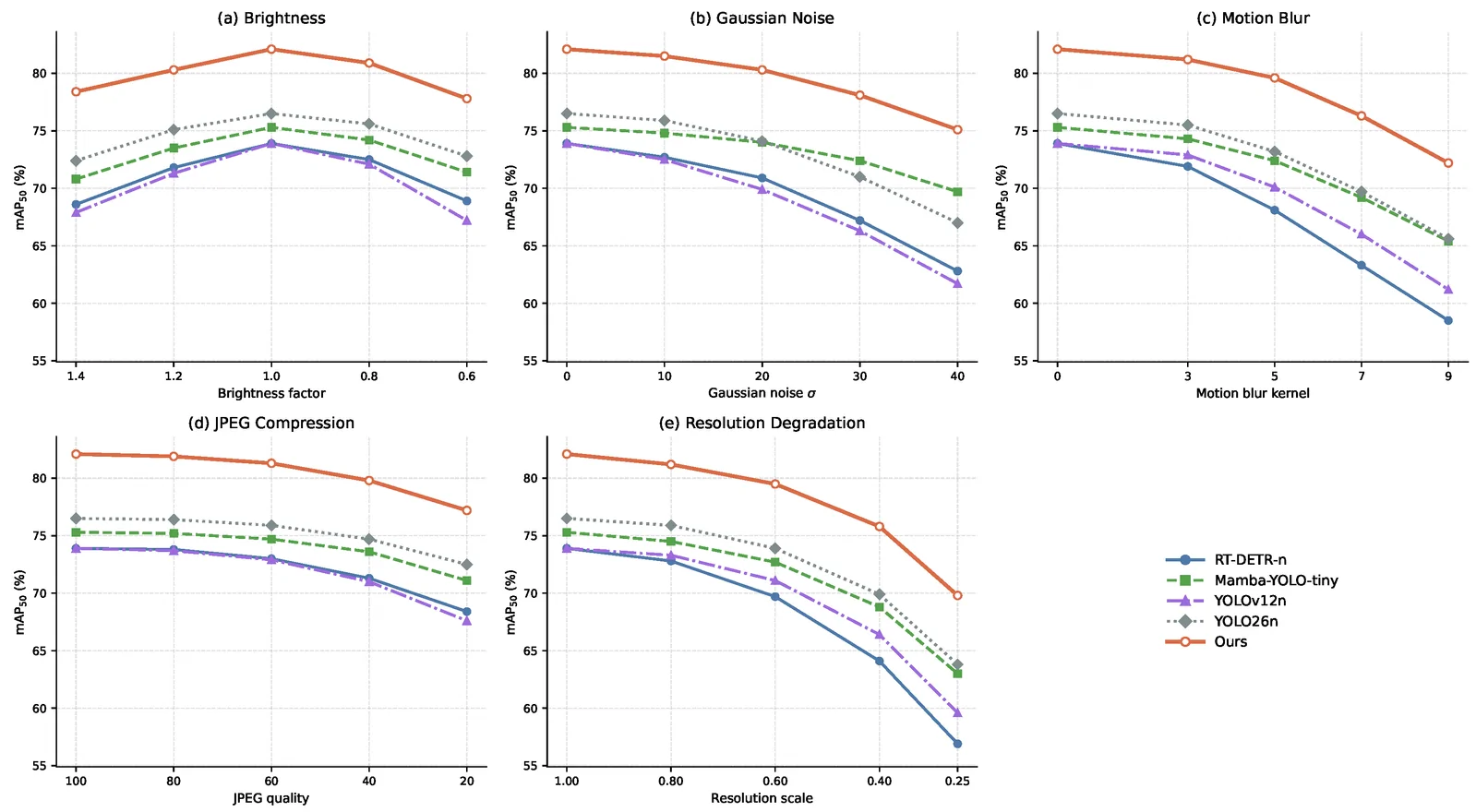
Robustness comparison under (**a**) brightness variation, (**b**) Gaussian noise, (**c**) motion blur, (**d**) JPEG compression, and (**e**) resolution degradation.

The robustness results are shown in Figure 13.

Table 22 presents absolute mAP50 and the performance decrease in percentage points relative to each model’s own clean reference, at every evaluated corruption setting. JPEG quality 100 is used as the clean-reference setting within the JPEG experiment.

As shown in Figure 13, all detectors exhibit performance degradation as image corruption becomes more severe, although the degradation patterns differ substantially across perturbations. Brightness variation and JPEG compression have relatively limited effects, whereas motion blur and resolution loss lead to more pronounced reductions because they directly weaken fine-grained behavioral cues.

MCHIME-Det maintains the highest mAP_50_ across all evaluated conditions. Under strong Gaussian noise (σ=40), motion blur (k=9), and severe JPEG compression (quality = 20), it retains 75.1%, 72.2%, and 77.2% mAP_50_, respectively. The advantage becomes particularly evident under resolution degradation, where MCHIME-Det achieves 69.8% mAP_50_ at a scale of 0.25, exceeding YOLO26n by 6.0 percentage points.

MCHIME-Det retains the highest absolute mAP_50_ among the evaluated models under the tested corruptions. Table 22 separately reports clean-relative degradation for each model, enabling comparison with its own clean-image reference. Severe blur and resolution loss produce the largest reductions and remain challenging for classroom behavior detection.

The synthetic perturbations measure sensitivity to the specified still-image corruption types and severity levels. Real classroom video can add time-varying camera motion, automatic exposure changes, and combinations of acquisition artifacts beyond this controlled protocol. At brightness factor 0.6, Gaussian-noise standard deviation 40, motion-blur kernel size 9, JPEG quality 20, and resolution scale 0.25, MCHIME-Det records mAP_50_ values of 77.8%, 75.1%, 72.2%, 77.2%, and 69.8%, corresponding to clean-relative reductions of 4.3, 7.0, 9.9, 4.9, and 12.3 percentage points.

## 5. Discussion

### 5.1. Behavior Recognition Difficulty

**Figure 14:**
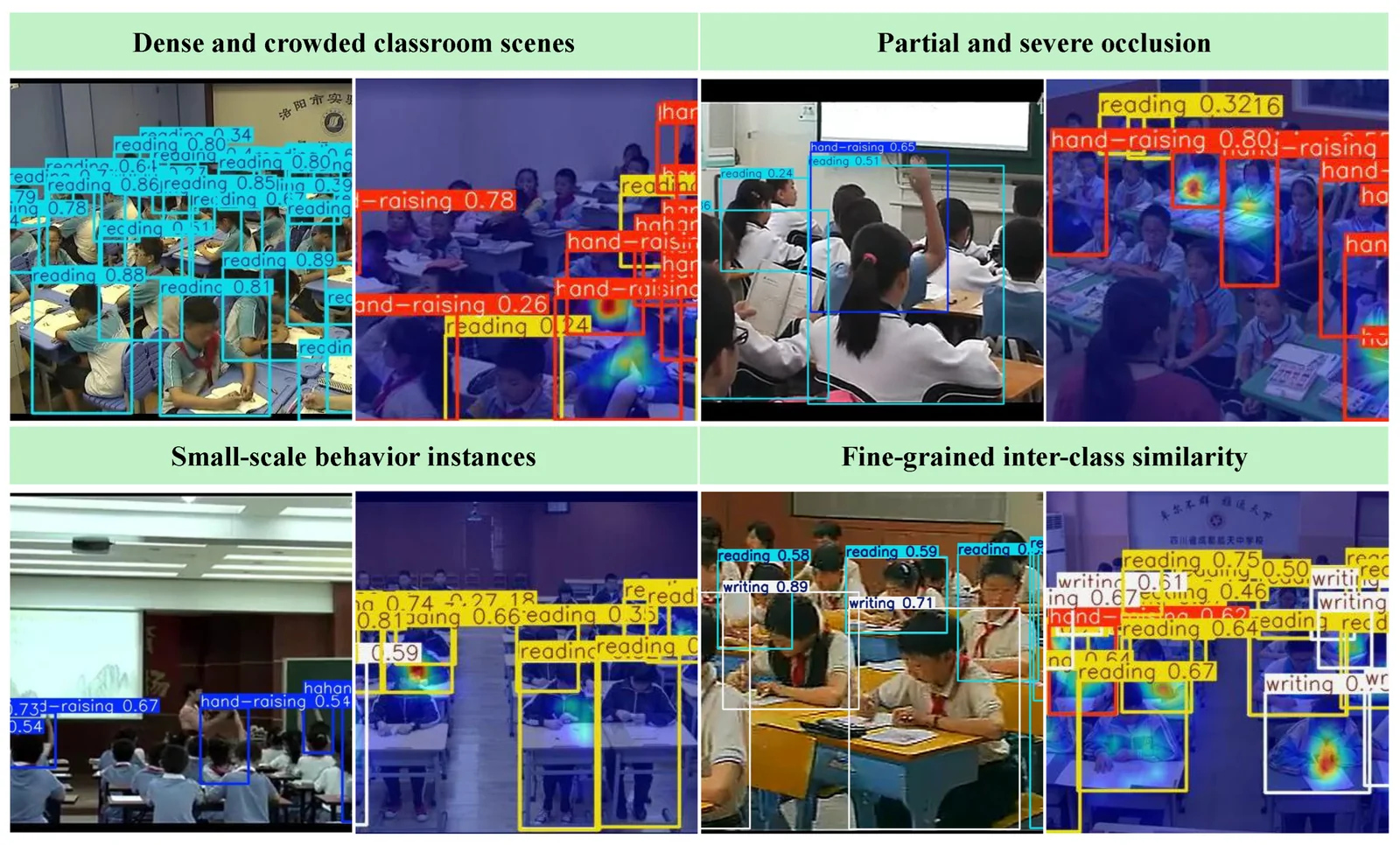
Representative difficult cases in classroom behavior recognition, including dense and crowded scenes, partial and severe occlusion, small-scale behavior instances, and fine-grained inter-class similarity.

Classroom behavior recognition is inherently more challenging than conventional object detection because behavioral categories are defined not only by object appearance but also by subtle differences in posture, hand–object interaction, and local spatial context. Systematic and task-specific studies similarly identify inter-class similarity, occlusion, small targets, and dense classroom layouts as recurrent challenges for vision-based classroom analysis [1,3,10,16]. Figure 14 illustrates four representative sources of difficulty observed in the test set: dense and crowded scenes, partial or severe occlusion, small-scale behavior instances, and fine-grained inter-class similarity.

In dense classroom scenes, multiple students frequently appear within a limited image region, producing closely spaced or overlapping instances. Occlusion further removes discriminative body and hand cues, making behavior recognition dependent on incomplete visual evidence. Small-scale instances introduce an additional difficulty because students located far from the camera contain only limited spatial details, particularly around the hands, books, and writing regions. These observations are consistent with the performance degradation identified in the preceding scale-, density-, and overlap-conditioned analyses. Comparable difficulties also arise in visually degraded sensing environments, where deep-learning-based image restoration has been integrated with object detection to recover weak target information before recognition [71]. A more fundamental challenge arises from the fine-grained similarity between behavior categories. As illustrated in Figure 14, reading and writing may share highly similar seated postures and surrounding objects, while their distinction often depends on subtle hand movements and hand–object relationships. The proposed C3k2PMM and CGHFF modules alleviate these difficulties by strengthening multi-scale feature extraction and cross-level information interaction, whereas C2MECM provides complementary semantic cues when local evidence is incomplete. Nevertheless, ambiguous reading–writing transitions, severe occlusion, and extremely small instances remain difficult, indicating that future classroom behavior recognition may benefit from incorporating temporal information and more explicit modeling of human–object interactions.

### 5.2. Implications for Automated Classroom Observation

Automated classroom behavior detection provides a scalable complement to traditional manual observation, which is often labor-intensive and difficult to sustain over large student populations. By detecting hand-raising, reading, and writing in individual frames, the proposed framework can provide frame-level inputs for structured behavioral records and longitudinal analysis. The measured edge inference throughput suggests potential for reduced-rate classroom activity summaries in a future end-to-end deployment.

These outputs can support quantitative analysis of observable participation patterns across students, lessons, and teaching settings. Detected hand-raising, reading, and writing are observable actions, not direct measurements of students’ cognitive states or learning outcomes; the same behavior can arise in different cognitive and instructional contexts. This distinction is consistent with recent reviews and cognitive-engagement studies that treat visual behavior as an observable indicator rather than a complete representation of internal learning state [1,2]. Automated observation is therefore best viewed as a supporting measurement tool rather than a substitute for teachers or human observers. Future applications should further consider temporal context and classroom interactions [72]. For deployments involving minors, appropriate institutional authorization and notice, data minimization, secure storage and transmission, restricted access to images and derived behavior records, and explicit retention and deletion periods are important safeguards. Authorized educators should retain human oversight of behavior summaries and review uncertain detections before any educational interpretation. Classroom layout, camera viewpoint, and student-population differences can also affect performance, making setting-specific evaluation important before operational use.

### 5.3. Failure Cases, Limitations, and Future Directions

**Figure 15:**
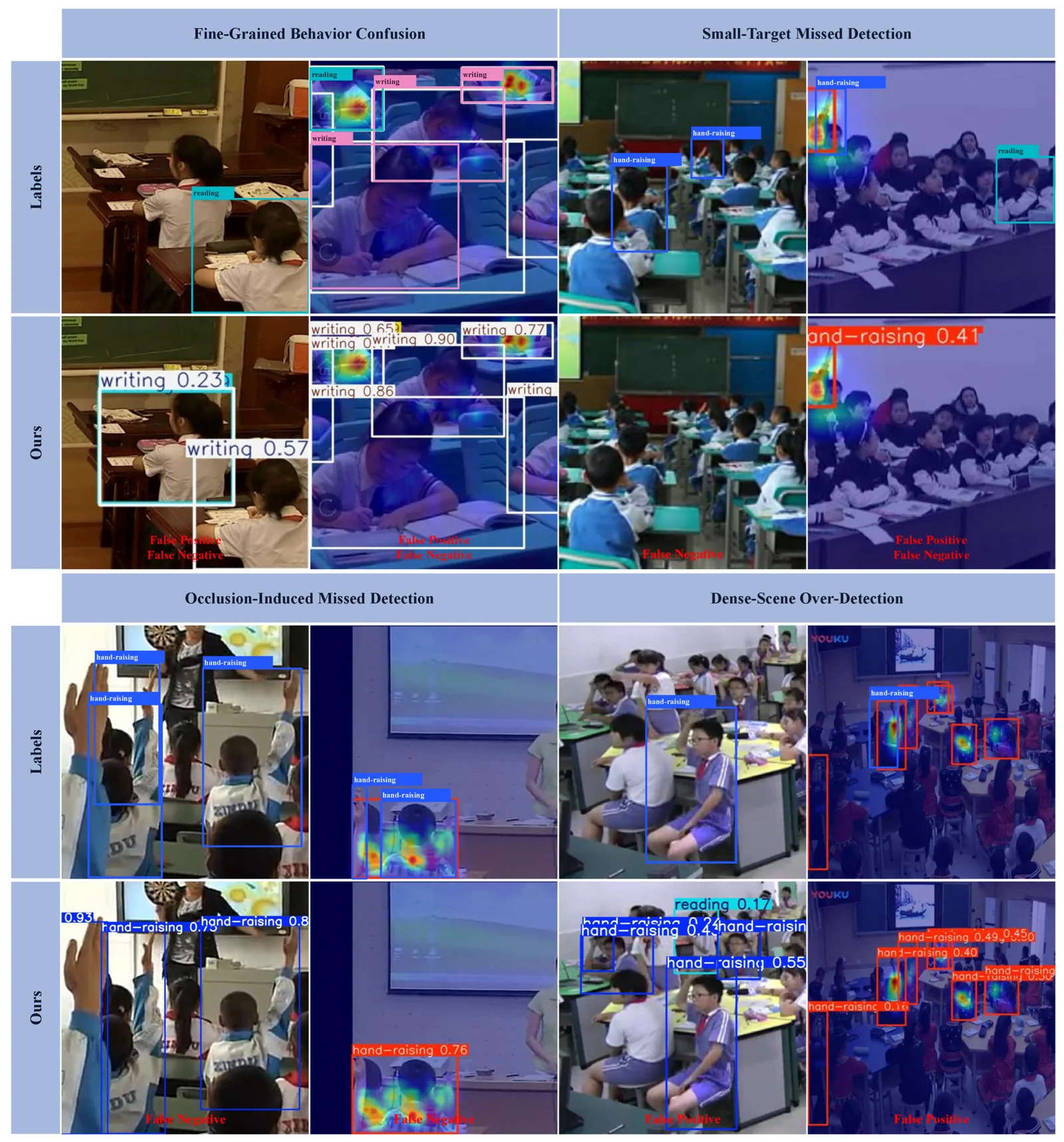
Representative failure cases of MCHIME-Det, including fine-grained behavior confusion, small-target missed detection, occlusion-induced missed detection, and dense-scene over-detection. Ground-truth annotations and model predictions are shown for comparison.

Although MCHIME-Det improves detection accuracy on the reported benchmark and maintains competitive performance under the tested synthetic corruptions, several challenging cases remain, as illustrated in Figure 15. The representative examples reveal four major failure modes: fine-grained behavior confusion, small-target missed detection, occlusion-induced missed detection, and dense-scene over-detection.

Fine-grained confusion mainly occurs between reading and writing, whose visual differences are often restricted to subtle hand movements and hand–object relationships. Small or distant students may also be missed when behaviorally relevant regions occupy only a few pixels. Under severe occlusion, incomplete body and hand cues can result in false negatives, whereas highly crowded scenes may produce redundant responses or false positives because of closely adjacent and overlapping instances. These cases indicate that, despite improved multi-scale and contextual representation, reliable recognition remains difficult when discriminative visual evidence is insufficient or ambiguous.

These observations also highlight several limitations of the current framework. First, the detector operates on individual frames and therefore cannot exploit temporal cues that may help distinguish visually similar or transitional behaviors. Second, extremely small and heavily occluded instances remain sensitive to information loss. The overlap-conditioned analysis quantifies geometric bounding-box overlap, while this failure mode concerns the physically visible evidence available for heavily occluded students. Third, the current three-class taxonomy captures only a subset of classroom activities, and performance may vary under classroom layouts and imaging conditions beyond those represented in the training data. Finally, the current split is image-level, and sequence identifiers needed for temporal separation are absent from the released metadata. The public sources may contain visually similar or adjacent classroom frames. A future sequence- or classroom-grouped evaluation would therefore provide a stronger test of temporal generalization.

Future work could therefore incorporate temporal behavior modeling and more explicit human–object interaction reasoning to reduce ambiguity between fine-grained classes. Improving feature preservation for very small instances and introducing stronger occlusion-aware or crowded-scene reasoning may further reduce missed and redundant detections. Super-resolution-assisted detection, which has been explored for small targets in low-quality remote sensing imagery, may provide a complementary direction for recovering fine-scale structural cues [73,74]. Extending the behavior taxonomy and evaluating the framework across more diverse classroom environments would also support future deployment assessment.

## 6. Conclusions

This study presented MCHIME-Det, a lightweight classroom behavior detector that integrates C3k2PMM, CGHFF, and C2MECM to address weak local action cues, cross-level semantic interference, and ambiguous high-level representations. On the combined two-source public dataset of 11,879 images and 71,657 annotated instances, the model achieved 82.1% mAP_50_ and 61.4% mAP_50:95_, representing gains of 5.6 and 4.4 percentage points over YOLO26n while retaining 2.36 M parameters, 5.2 GFLOPs, and 182 FPS on the RTX 4090 platform.

Beyond overall accuracy, the proposed modules remained effective across three additional lightweight YOLO backbones and showed larger relative benefits for small targets, high-density scenes, and instances with heavy geometric bounding-box overlap. Severe physical occlusion nevertheless remains a failure mode. The zero-shot evaluation provides the primary cross-source transfer evidence across the three retained external subsets, whereas dataset-specific retraining characterizes adaptation on held-out images within each sampled source. Corruption and cross-hardware experiments characterized performance under visual degradation and heterogeneous deployment hardware. These results suggest that complementary local–global spatial modeling, selective hierarchical fusion, and memory-assisted compensation can improve difficult-scene classroom behavior detection without relying on model expansion. Remaining errors under severe occlusion, extreme resolution loss, and reading–writing ambiguity indicate that future work should incorporate temporal cues, explicit human–object interaction reasoning, and broader behavior taxonomies while preserving the lightweight inference objective.

## Figures and Tables

**Table 1:** Image composition of the classroom behavior dataset from two public sources after data curation.

Data Source	Source Type	Images	Proportion
SCB-2025-7-public-1	Public	6864	57.8%
SCB-Dataset3-S-2	Public	5015	42.2%
Total	—	11,879	100%

**Table 2:** Statistics of the combined public classroom behavior dataset.

Subset	Images	Hand-Raising	Reading	Writing	Instances
Train	9503	19,580	27,101	10,275	56,956
Validation	1187	2575	3623	1307	7505
Test	1189	2504	3358	1334	7196
Total	11,879	24,659	34,082	12,916	71,657

**Table 3:** Experimental environment.

Item	Configuration
Operating system	Windows 11
CPU	Intel Core Ultra 9 285K (3.70 GHz)
RAM	64 GB
GPU	NVIDIA GeForce RTX 4090
GPU memory	24 GB
Deep learning framework	PyTorch 2.1.0
CUDA version	11.8
Python version	3.10.20

**Table 4:** Training settings for YOLO26n and its variants.

Parameter	Value
Input image size	640×640
Epochs	200
Batch size	16
Optimizer	SGD
Initial learning rate ($lr_{0}$)	0.001
Final learning-rate factor (lrf)	0.01
Momentum	0.937
Weight decay	0.0005
Workers	8

**Table 5:** Inference-speed benchmarking settings.

Parameter	Value
Input image size	640×640 (SSD300: 300×300)
Batch size	1
Warm-up iterations	200
Timed iterations	1000
Precision	FP32
Model fusion	Enabled
Gradient computation	Disabled

**Table 6:** Comparison with representative object detectors on the classroom behavior dataset. Selected mAP results are reported as mean and SD across independent runs.

Model	P (%)	R (%)	mAP_50_ (%)	mAP_50:95_ (%)
RT-DETR-n [43]	67.4	68.9	73.9	53.8
GhostNet-n	68.1	66.8	73.3	52.7
Mamba-YOLO-tiny [44]	71.7	68.7	75.3	56.0
SSD300-MobileNetV2 [45,46]	65.1	65.1	70.2	49.7
Faster R-CNN (ResNet-50) [47,48]	64.2	66.4	70.8	49.8
YOLO-Worldv2-n [49]	69.2	68.9	74.7	55.2
YOLOv9t [50]	66.6	65.8	71.5	52.5
YOLOv10n [51]	67.2	66.8	72.7	51.1
YOLOv11n [52]	65.1	67.2	71.5	51.5
YOLOv12n [53]	68.3	68.3	73.9	53.2
YOLOv13n [54]	67.5	66.5	73.0	51.6
YOLO26n [55]	68.7	71.2	76.5±0.31	57.0±0.39
Classroom-specific behavior detectors				
CBPH-Net [14]	71.4	71.9	77.8	56.5
SLBDetection-Net(Swin) [15]	73.6	73.5	$\underline{79.40\pm 0.55}$	$\underline{58.40\pm 0.65}$
**Ours**	**76.3**	**74.9**	82.1±0.21	61.4±0.27

Note: Bold values indicate the best performance, while underlined values indicate the second-best performance among all compared methods. CBPH-Net and SLBDetection-Net are specifically designed for classroom behavior detection. Only the mAP results for YOLO26n, SLBDetection-Net (Swin), and MCHIME-Det include SDs from independent runs; the remaining cells report point estimates.

**Table 7:** Ablation results of different combinations of the proposed modules. Accuracy metrics are reported as mean ± standard deviation over five independent runs.

Model	C3k2PMM	CGHFF	C2MECM	P (%)	R (%)	mAP_50_ (%)	mAP_50:95_ (%)	Params (M)	FLOPs (G)	Size (MB)	FPS
M0	–	–	–	68.7±0.43	71.2±0.58	76.5±0.31	57.0±0.39	2.37	5.2	5.1	190
M1	✓	–	–	70.8±0.37	71.0±0.49	77.4±0.27	57.8±0.34	2.11	4.8	4.9	188
M2	–	✓	–	73.6±0.46	70.7±0.55	78.9±0.33	58.5±0.41	2.60	5.5	5.3	186
M3	–	–	✓	70.1±0.52	73.4±0.44	78.1±0.36	58.9±0.29	2.39	5.3	5.1	188
M12	✓	✓	–	$\underline{75.9\pm 0.39}$	72.2±0.47	80.1±0.24	59.7±0.32	2.34	5.1	5.1	184
M13	✓	–	✓	72.6±0.34	73.7±0.51	79.4±0.28	60.1±0.36	2.13	4.9	4.9	186
M23	–	✓	✓	74.6±0.41	$\underline{74.3\pm 0.43}$	$\underline{80.7\pm 0.26}$	$\underline{60.6\pm 0.38}$	2.62	5.6	5.3	184
M123	✓	✓	✓	76.3±0.35	74.9±0.48	82.1±0.21	61.4±0.27	2.36	5.2	5.1	182

Note: Bold values indicate the best performance, while underlined values indicate the second-best performance among the compared variants. Params, GFLOPs, model size, and FPS are reported as single values.

**Table 8:** Internal component ablation results for C3k2PMM, CGHFF, and C2MECM. Accuracy metrics are reported as mean and standard deviation over five independent runs. Bold values indicate the best performance within each module group.

Module	Model	C3k2PMM Components			CGHFF Components		C2MECM Components		Performance	
		Multi-Scale Pyramid	SS2D Scanning	Directional Masking	GCSA	Reciprocal Modulation	SCStream	Multi-Expert Routing	mAP_50_ (%)	mAP_50:95_ (%)
Baseline	M0	–	–	–	–	–	–	–	76.5±0.31	57.0±0.39
C3k2PMM	M1-a	✓	–	–	–	–	–	–	76.9±0.39	57.3±0.47
	M1-b	–	✓	✓	–	–	–	–	76.7±0.46	57.6±0.32
	M1-c	✓	✓	–	–	–	–	–	77.1±0.37	57.5±0.29
	M1	✓	✓	✓	–	–	–	–	77.4±0.27	57.8±0.34
CGHFF	M2-a	–	–	–	–	✓	–	–	78.2±0.42	57.9±0.48
	M2-b	–	–	–	✓	–	–	–	77.5±0.28	58.0±0.35
	M2	–	–	–	✓	✓	–	–	78.9±0.33	58.5±0.41
C2MECM	M3-a	–	–	–	–	–	–	✓	77.4±0.45	58.3±0.38
	M3-b	–	–	–	–	–	✓	–	77.8±0.34	58.6±0.31
	M3	–	–	–	–	–	✓	✓	78.1±0.36	58.9±0.29

**Table 9:** Cross-architecture transferability of C3k2PMM, CGHFF, and C2MECM. “+All” denotes the joint integration of the three proposed modules. Baseline and “+All” values are mean and SD over five independent runs; the intermediate single-module variants are point estimates.

Architecture	Variant	mAP_50_ (%)	mAP_50:95_ (%)	Params (M)
YOLOv10n	Baseline	72.2±0.36	51.3±0.43	2.26
	+C3k2PMM	73.4	51.8	2.03
	+CGHFF	74.6	52.6	2.48
	+C2MECM	74.1	52.7	2.28
	+All	76.4±0.44	54.4±0.32	2.27
YOLOv12n	Baseline	73.3±0.28	53.1±0.35	2.55
	+C3k2PMM	74.8	54.0	2.32
	+CGHFF	75.9	54.7	2.76
	+C2MECM	75.4	55.2	2.58
	+All	78.4±0.39	56.4±0.48	2.56
YOLOv13n	Baseline	73.2±0.46	51.2±0.30	2.46
	+C3k2PMM	74.0	52.5	2.24
	+CGHFF	75.5	53.1	2.67
	+C2MECM	74.8	53.6	2.49
	+All	77.0±0.33	55.2±0.41	2.47

**Table 10:** Behavior-wise AP_50_ comparison between YOLO26n and the proposed model. Gain denotes the improvement over YOLO26n in percentage points.

Behavior	YOLO26n AP_50_ (%)	Ours AP_50_ (%)	Gain (pp)
Hand-raising	76.6	83.9	+7.3
Reading	74.7	79.2	+4.5
Writing	78.2	83.1	+4.9
Mean	76.5	82.1	+5.6

**Table 11:** Definition and instance statistics of the scale-conditioned subsets.

Scale Group	Criterion	Train	Validation	Test
Small	ρ<1.2%	13,663	2002	1776
Medium	1.2%≤ρ<2.7%	14,775	1946	1878
Large	2.7%≤ρ<5.9%	14,185	1822	1787
Very Large	ρ≥5.9%	14,333	1735	1755
Total	–	56,956	7505	7196

**Table 12:** Instance-level scale-conditioned mAP_50_ (%) comparison of representative detectors. Scale Gap denotes the performance difference between Very Large and Small targets, while Small Gain denotes the improvement over YOLO26n for Small targets.

Model	Overall	Small	Medium	Large	Very Large	Scale Gap	Small Gain
RT-DETR-n	73.9	63.8	71.2	76.9	80.2	16.4	−3.3
SSD300-MobileNetV2	70.2	61.6	67.7	73.4	77.0	15.4	−5.5
Mamba-YOLO-tiny	75.3	66.4	73.6	77.8	81.0	14.6	−0.7
YOLO-Worldv2-n	74.7	66.9	72.4	77.3	79.8	12.9	−0.2
YOLOv12n	73.9	64.8	71.6	76.7	80.0	15.2	−2.3
YOLO26n	76.5	67.1	74.5	79.4	81.3	14.2	0.0
**Ours**	**82.1**	**75.4**	**80.7**	**84.0**	**85.8**	**10.4**	**+8.3**

Note: Bold and underlined values indicate the best and second-best results, respectively. A lower Scale Gap is preferred. Small Gain is calculated relative to YOLO26n.

**Table 13:** Definition and image statistics of the density-conditioned subsets.

Density Group	Criterion	Train	Validation	Test
Low	D≤2	3580	437	446
Medium	3≤D≤6	3210	400	416
High	D≥7	2713	350	327
Total	–	9503	1187	1189

**Table 14:** Density-conditioned mAP_50_ (%) comparison of representative detectors. Density Drop denotes the performance decrease from Low- to High-density scenes, while High Gain denotes the improvement over YOLO26n in the High-density subset.

Model	Overall	Low	Medium	High	Density Drop	High Gain
RT-DETR-n	73.9	78.4	74.1	67.2	11.2	−3.6
SSD300-MobileNetV2	70.2	75.6	69.8	62.9	12.7	−7.9
Mamba-YOLO-tiny	75.3	79.6	75.2	69.1	10.5	−1.7
YOLO-Worldv2-n	74.7	78.7	74.6	69.8	8.9	−1.0
YOLOv12n	73.9	78.1	73.8	67.5	10.6	−3.3
YOLO26n	76.5	80.5	76.4	70.8	9.7	0.0
**Ours**	**82.1**	**84.8**	**82.4**	**78.0**	**6.8**	**+7.2**

Note: Bold and underlined values indicate the best and second-best results, respectively. A lower Density Drop is preferred. High Gain is calculated relative to YOLO26n.

**Table 15:** Definition and instance statistics of the overlap-conditioned subsets.

Overlap Group	Criterion	Train	Validation	Test
Low	O<0.05	14,290	1801	1762
Mild	0.05≤O<0.30	14,498	2038	1751
Moderate	0.30≤O<0.50	14,296	1884	1894
Heavy	O≥0.50	13,872	1782	1789
Total	–	56,956	7505	7196

**Table 16:** Instance-level overlap-conditioned mAP_50_ (%) comparison of representative detectors. Overlap Drop denotes the performance decrease from Low to Heavy overlap, while Heavy Gain denotes the improvement over YOLO26n in the Heavy-overlap subset.

Model	Overall	Low	Mild	Moderate	Heavy	Overlap Drop	Heavy Gain
RT-DETR-n	73.9	80.2	75.8	72.0	65.9	14.3	−4.0
SSD300-MobileNetV2	70.2	76.8	71.9	67.1	61.7	15.1	−8.2
Mamba-YOLO-tiny	75.3	80.6	76.7	73.4	68.5	12.1	−1.4
YOLO-Worldv2-n	74.7	79.8	76.5	73.7	69.4	10.4	−0.5
YOLOv12n	73.9	79.4	75.0	71.6	66.8	12.6	−3.1
YOLO26n	76.5	81.7	77.9	74.6	69.9	11.8	0.0
**Ours**	**82.1**	**85.5**	**83.0**	**80.4**	**76.9**	**8.6**	**+7.0**

Note: Bold and underlined values indicate the best and second-best results, respectively. A lower Overlap Drop is preferred. Heavy Gain is calculated relative to YOLO26n.

**Table 17:** External classroom behavior datasets used for cross-source evaluation, with category-specific annotation counts for each 1000-image sample.

Dataset	Available Images	Selected Images	Original Classes	Hand-Raising Instances	Reading Instances	Writing Instances	Total Instances
STBD-08 [14]	8884	1000	8	451	7296	7670	15,417
Student Behaviour Detection [61]	2471	1000	12	869	2164	404	3437
Student Classroom Behavior Dataset [62]	2032	1000	6	1176	6948	2572	10,696

Note: The retained classes are hand-raising, reading, and writing. Each dataset contributes 1000 sampled images after category harmonization and duplicate screening. The class counts denote annotated bounding-box instances, not numbers of images, and the original classes column refers to the source dataset taxonomy.

**Table 18:** Zero-shot cross-source performance on three external classroom behavior datasets (mAP_50_, %). Each source contributes 1000 images; dataset-level values are mean and SD across five independent runs.

Dataset	YOLOv13n	YOLO26n	Best Other	Ours	Δ
STBD-08	66.7±1.4	$\underline{69.5\pm 1.2}$	68.6±1.5	72.2±1.3	+2.7
SBD	63.2±2.0	65.8±1.8	$\underline{66.5\pm 1.9}$	69.3±2.1	+2.8
SCBD	61.0±1.7	63.9±1.6	$\underline{64.3\pm 1.8}$	67.6±1.5	+3.3
Mean	63.6	66.4	66.5	**69.7**	+3.2

Note: Bold and underlined values indicate the best and second-best results, respectively. Best Other denotes the highest-performing comparison detector excluding YOLO26n and Ours. For each dataset, Δ denotes the improvement of Ours over the stronger of YOLO26n and Best Other; in the Mean row, it denotes the difference between the mean of Ours and the larger of the two comparator means. The mean-row difference is calculated before rounding the means for display. Dataset-level entries report mean and SD across five independent runs; the Mean row averages the three dataset-level means and does not represent a pooled SD.

**Table 19:** Cross-source retraining performance on three external classroom behavior datasets (mAP_50_, %). Each 1000-image subset uses the same fixed seed-42 700/100/200 train/validation/test split; dataset-level values are mean and SD across five independent runs.

Dataset	YOLOv13n	YOLO26n	Best Other	Ours	Δ
STBD-08	77.7±1.6	$\underline{80.2\pm 0.7}$	79.9±1.8	82.6±1.6	+2.4
SBD	75.2±2.6	77.8±1.4	$\underline{78.7\pm 1.2}$	81.0±1.5	+2.3
SCBD	73.9±2.1	76.4±1.9	$\underline{77.0\pm 2.3}$	79.6±1.7	+2.6
Mean	75.6	78.1	78.5	**81.1**	+2.5

Note: Bold and underlined values indicate the best and second-best results, respectively. Best Other denotes the highest-performing comparison detector excluding YOLO26n and Ours. For each dataset, Δ denotes the improvement of Ours over the stronger of YOLO26n and Best Other; in the Mean row, it denotes the difference between the mean of Ours and the larger of the two comparator means. The mean-row difference is calculated before rounding the means for display. Dataset-level entries report mean and SD across five independent runs; the Mean row averages the three dataset-level means and does not represent a pooled SD.

**Table 20:** Comparison of model complexity and inference efficiency with representative general-purpose and classroom-specific detectors.

Model	Params (M)	GFLOPs	Size (MB)	FPS
RT-DETR-n	16.76	42.6	34.1	74
GhostNet-n	**1.71**	5.0	**3.8**	**191**
Mamba-YOLO-tiny	6.02	13.6	12.3	79
SSD300-MobileNetV2	3.69	**1.4**	14.8	184
Faster R-CNN (ResNet-50)	28.33	94.8	108.0	48
YOLO-Worldv2-n	3.53	9.6	7.4	169
YOLOv9t	1.97	7.6	4.7	80
YOLOv10n	2.26	6.5	5.8	171
YOLOv11n	2.58	6.3	5.5	169
YOLOv12n	2.55	6.3	5.5	140
YOLOv13n	2.46	6.2	5.4	99
YOLO26n	2.37	5.2	5.1	190
Classroom-specific behavior detectors				
CBPH-Net	6.58	16.10	26.57	112
SLBDetection-Net (Swin)	58.00	208.9	116.7	76
**Ours**	2.36	5.2	5.1	182

Note: Params and GFLOPs characterize model complexity, whereas Size (MB) denotes serialized checkpoint size on disk, which also depends on weight-storage precision. FPS represents forward-pass throughput under the tested input resolution. Bold and underlined values denote the best and second-best results, respectively. The FPS of CBPH-Net was measured in our implementation under the same benchmarking protocol described in Table 5.

**Table 21:** Perturbation settings for robustness evaluation.

Perturbation	Evaluated Settings	Clean Reference
Brightness factor	1.4, 1.2, 1.0, 0.8, 0.6	1.0
Gaussian noise (σ)	0, 10, 20, 30, 40	0
Motion blur kernel	0, 3, 5, 7, 9	0
JPEG quality	100, 80, 60, 40, 20	100
Resolution scale	1.00, 0.80, 0.60, 0.40, 0.25	1.00

**Table 22:** Absolute mAP50 under five synthetic corruptions and the decrease relative to each model’s clean reference (percentage points). Each cell shows mAP50 and the downward-arrow clean-relative drop; bold values denote the highest absolute accuracy at each setting.

Setting	RT-DETR-n	Mamba-YOLO-tiny	YOLOv12n	YOLO26n	MCHIME-Det
Brightness factor					
1.4	68.6 (↓5.3)	70.8 (↓4.5)	67.9 (↓6.0)	72.4 (↓4.1)	**78.4 (↓3.7)**
1.2	71.8 (↓2.1)	73.5 (↓1.8)	71.3 (↓2.6)	75.1 (↓1.4)	**80.3 (↓1.8)**
1.0 (Clean)	73.9 (↓0.0)	75.3 (↓0.0)	73.9 (↓0.0)	76.5 (↓0.0)	**82.1 (↓0.0)**
0.8	72.5 (↓1.4)	74.2 (↓1.1)	72.1 (↓1.8)	75.6 (↓0.9)	**80.9 (↓1.2)**
0.6	68.9 (↓5.0)	71.4 (↓3.9)	67.2 (↓6.7)	72.8 (↓3.7)	**77.8 (↓4.3)**
Gaussian noise (σ)					
0 (Clean)	73.9 (↓0.0)	75.3 (↓0.0)	73.9 (↓0.0)	76.5 (↓0.0)	**82.1 (↓0.0)**
10	72.7 (↓1.2)	74.8 (↓0.5)	72.5 (↓1.4)	75.9 (↓0.6)	**81.5 (↓0.6)**
20	70.9 (↓3.0)	74.0 (↓1.3)	69.9 (↓4.0)	74.1 (↓2.4)	**80.3 (↓1.8)**
30	67.2 (↓6.7)	72.4 (↓2.9)	66.3 (↓7.6)	71.0 (↓5.5)	**78.1 (↓4.0)**
40	62.8 (↓11.1)	69.7 (↓5.6)	61.7 (↓12.2)	67.0 (↓9.5)	**75.1 (↓7.0)**
Motion blur (*k*)					
0 (Clean)	73.9 (↓0.0)	75.3 (↓0.0)	73.9 (↓0.0)	76.5 (↓0.0)	**82.1 (↓0.0)**
3	71.9 (↓2.0)	74.3 (↓1.0)	72.9 (↓1.0)	75.5 (↓1.0)	**81.2 (↓0.9)**
5	68.1 (↓5.8)	72.4 (↓2.9)	70.1 (↓3.8)	73.2 (↓3.3)	**79.6 (↓2.5)**
7	63.3 (↓10.6)	69.2 (↓6.1)	66.0 (↓7.9)	69.7 (↓6.8)	**76.3 (↓5.8)**
9	58.5 (↓15.4)	65.4 (↓9.9)	61.2 (↓12.7)	65.6 (↓10.9)	**72.2 (↓9.9)**
JPEG quality (*Q*)					
100 (Clean ref.)	73.9 (↓0.0)	75.3 (↓0.0)	73.9 (↓0.0)	76.5 (↓0.0)	**82.1 (↓0.0)**
80	73.8 (↓0.1)	75.2 (↓0.1)	73.7 (↓0.2)	76.4 (↓0.1)	**81.9 (↓0.2)**
60	73.0 (↓0.9)	74.7 (↓0.6)	72.9 (↓1.0)	75.9 (↓0.6)	**81.3 (↓0.8)**
40	71.3 (↓2.6)	73.6 (↓1.7)	71.0 (↓2.9)	74.7 (↓1.8)	**79.8 (↓2.3)**
20	68.4 (↓5.5)	71.1 (↓4.2)	67.6 (↓6.3)	72.5 (↓4.0)	**77.2 (↓4.9)**
Resolution scale					
1.00 (Clean)	73.9 (↓0.0)	75.3 (↓0.0)	73.9 (↓0.0)	76.5 (↓0.0)	**82.1 (↓0.0)**
0.80	72.8 (↓1.1)	74.5 (↓0.8)	73.3 (↓0.6)	75.9 (↓0.6)	**81.2 (↓0.9)**
0.60	69.7 (↓4.2)	72.7 (↓2.6)	71.1 (↓2.8)	73.9 (↓2.6)	**79.5 (↓2.6)**
0.40	64.1 (↓9.8)	68.8 (↓6.5)	66.4 (↓7.5)	69.9 (↓6.6)	**75.8 (↓6.3)**
0.25	56.9 (↓17.0)	63.0 (↓12.3)	59.6 (↓14.3)	63.8 (↓12.7)	**69.8 (↓12.3)**

## Data Availability

The publicly available datasets used in this study can be accessed through the sources cited in Section 2.1, and the author-generated annotations are available from the corresponding authors upon reasonable request.
